# Caspase‐6 Controls Lipid and Energy Metabolism in Diet‐Induced Obesity

**DOI:** 10.1002/advs.202514784

**Published:** 2026-04-13

**Authors:** Abhishek Gupta, Wenjing You, Linmeng Han, Jianfei Ji, Meixia Pan, Xianlin Han, Xiaoli Sun, Peng Zhao

**Affiliations:** ^1^ Department of Biochemistry and Structural Biology University of Texas Health Science Center San Antonio San Antonio Texas USA; ^2^ Department of Pharmacology University of Texas Health Science Center San Antonio San Antonio Texas USA; ^3^ Barshop Institute for Longevity and Aging Studies University of Texas Health Science Center San Antonio San Antonio Texas USA; ^4^ Mays Cancer Center University of Texas Health Science Center San Antonio San Antonio Texas USA; ^5^ Transplant Center University of Texas Health Science Center San Antonio San Antonio Texas USA

**Keywords:** adipose tissue, ATGL, caspase‐6, lipid metabolism, obesity

## Abstract

Caspases are cysteine proteases that regulate programmed cell death. While caspase‐6, an executioner caspase, is known for its role in neurodegeneration and cell death, its broader physiological functions remain poorly understood. Our previous study revealed that caspase‐6 drives liver injury and fibrosis in metabolic dysfunction‐associate steatohepatitis. Here, we report that caspase‐6 deficiency protects against high fat diet‐induced obesity. Both global and adipocyte‐specific caspase‐6 knockout mice exhibit increased energy expenditure, reduced adiposity and inflammation, and improved glucose metabolism. Mechanistically, caspase‐6 directly cleaves peroxisome proliferator‐activated receptor gamma (PPARγ) and its cofactor specificity protein 1 (SP1), thereby suppressing adipose triglyceride lipase (ATGL) expression. Caspase‐6 deficiency restores ATGL, enhancing lipolysis and elevating fatty acyl esters of hydroxy fatty acids (FAHFAs), which alleviate inflammation and enhance insulin sensitivity. These findings uncover a novel Casp6‐PPARγ/SP1‐ATGL axis in adipose tissue and establish caspase‐6 as a potential therapeutic target for obesity and insulin resistance.

## Graphical abstract

This study reveals an unknown metabolic role of caspase‐6 beyond cell death. Caspase‐6 deficiency protects against HFD‐induced obesity by increasing energy expenditure, reducing inflammation, and improving glucose metabolism. Mechanistically, caspase‐6 cleaves PPARγ and SP1 to suppress ATGL expression. Caspase‐6 deficiency restores ATGL‐driven lipolysis and FAHFA production. These findings identify a novel Casp6‐PPARγ/SP1‐ATGL axis and position caspase‐6 as a therapeutic target.

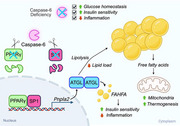

## 1 Introduction

Caspases are a family of cysteine proteases that play a pivotal role in programmed cell death [1]. Based on their functions, caspases are categorized into two classes: initiator and executioner caspases. Initiator caspases, including caspase‐2, ‐8, ‐9, ‐10, are responsible for the activation of executioners and the initiation of apoptotic cascade, whereas the executioner caspases, including caspase‐3, ‐6, ‐7, directly cleave cell structural components to cause cell death [1]. As a lesser studied family member, caspase‐6 is known for its pathogenic role in neurodegenerative diseases, such as Huntington's disease and Alzheimer's disease [2]. Our recent study revealed that caspase‐6 mediates hepatocellular death and liver injury in metabolic dysfunction‐associated steatohepatitis (MASH) [3], highlighting its involvement in metabolic diseases. However, it remains unknown whether caspase‐6 plays a role in the disruption of metabolic homeostasis.

Obesity has emerged as a global epidemic, primarily driven by a disruption of energy homeostasis caused by the imbalance between energy intake and energy expenditure [4, 5]. Adipose tissue plays a crucial role in maintaining systemic energy balance by regulating glucose and lipid metabolism [6, 7, 8, 9]. Adipose triglyceride lipase (ATGL) is a key enzyme catalyzing the breakdown of triglycerides, thereby reducing lipid storage in mature adipocytes [10, 11, 12]. Moreover, recent study demonstrated that ATGL catalyzes the synthesis of fatty acyl esters of hydroxy fatty acid (FAHFA), a class of lipids that improves glucose homeostasis and insulin sensitivity [13, 14, 15, 16, 17, 18]. Mechanistically, ATGL possesses transacylase activity, which allows it to transfer an acyl chain from di‐ and tri‐glyceride acyl donors to esterify hydroxy fatty acids [13]. FAHFAs, particularly palmitic‐acid‐hydroxy‐stearic acid (PAHSA), positively correlate with insulin sensitivity in humans. They have been shown to reduce proinflammatory cytokine production, enhance glucose stimulated insulin secretion, and promote GLP‐1 secretion, thus improving glucose metabolism and insulin sensitivity [14]. Consistently, overexpression of ATGL in adipose tissue has been shown to reduce triglyceride content, attenuate diet‐induced obesity, and enhance systemic insulin sensitivity [11]. Conversely, adipose‐specific deletion of ATGL exacerbates diet‐induced obesity and disrupts thermogenic capacity in brown adipose tissue [10]. Given the critical role of adipose ATGL in maintaining metabolic homeostasis, its activity is tightly regulated at both transcriptional and post‐translational levels [19, 20]. Transcriptional regulation of *Pnpla2* (the gene symbol of ATGL) involves a network of transcriptional factors, including peroxisome proliferator‐activated receptor gamma (PPARγ) and specificity protein 1 (SP1) [21, 22, 23]. PPARγ directly binds to the promoter of the *Pnpla2* gene and enhances its transcription, while SP1 functions as a co‐factor of PPARγ to upregulate *Pnpla2* expression in mature adipocytes [23]. In addition to regulating *Pnpla2* expression, PPARγ is a master regulator of metabolic processes, particularly those enhancing metabolic homeostasis [24, 25]. While stimulating adipogenesis in preadipocytes to increase lipid storage, PPARγ activation induces beiging of white adipocytes to promote catabolism and energy expenditure in high fat diet (HFD)‐fed mice [26, 27, 28, 29, 30]. It was shown that HFD feeding inhibits PPARγ activity in adipose tissues [31]. Thiazolidinediones (TZDs), a class of FDA‐approved synthetic ligands/agonists of PPARγ, leverage the beneficial effects of PPARγ to restore insulin sensitivity and glucose homeostasis in type 2 diabetes [27, 32, 33].

To further understand the role of caspase‐6 in metabolic disorders, we examined whether caspase‐6 contributes to metabolic dysregulation in diet‐induced obesity and insulin resistance. Our findings revealed that caspase‐6 expression is upregulated in a diet‐induced obesity mouse model and in human obesity. Knockout of caspase‐6 globally or in adipocytes improved energy metabolism and insulin sensitivity. Transcriptomic analysis indicated that caspase‐6 knockout increased *Pnpla2* (ATGL) expression in both white adipose tissues (WAT) and brown adipose tissue (BAT), while moderately increasing the expression of mitochondrial genes. Consistent with these results, lipidomic studies showed that caspase‐6 deficiency reduced triglycerides while increasing FAHFA levels in adipose tissue. Mechanistic studies uncovered that caspase‐6 directly cleaves both PPARγ and SP1 to regulate *Pnpla2* expression. Gene expression analysis of human datasets revealed a strong negative correlation between *Casp6* and PPARγ target genes. These discoveries not only enhance our understanding of the diverse functions of caspase‐6 beyond cell death regulation but also suggest potential therapeutic avenues for treating obesity and insulin resistance. As our previous study found that caspase‐6 is a potential therapeutic target in MASH [3], we now show that its inhibition can simultaneously improve obesity, diabetes, and MASH.

## 2 Results

### 2.1 Adipose Tissue Shows Elevated Caspase‐6 Expression in Obesity

**FIGURE 1:**
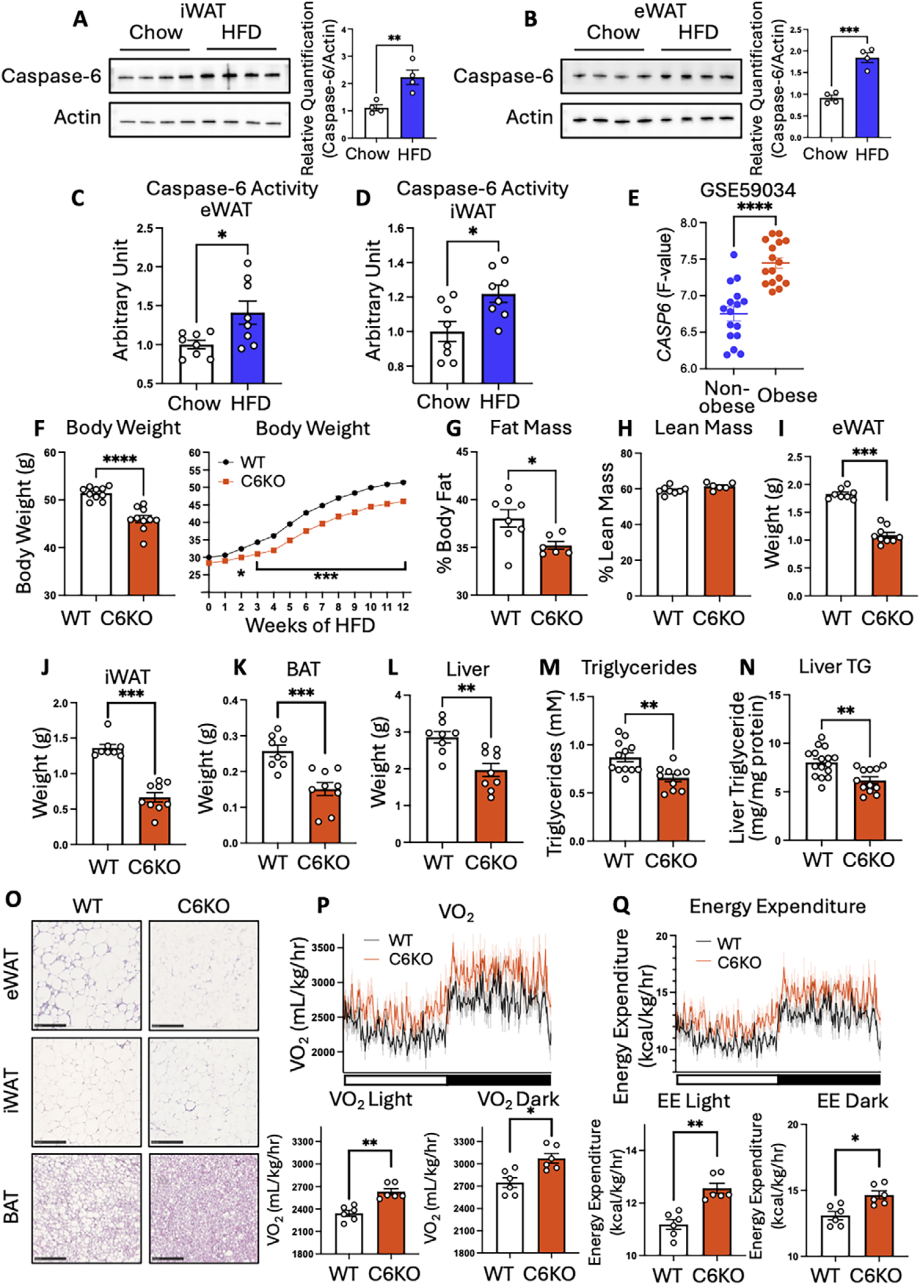
Caspase‐6 knockout attenuates diet‐induced obesity. (A, B). Immunoblot of caspase‐6 protein and quantification in iWAT (A) and eWAT (B) of C57BL/6J mice fed chow diet or 60% HFD for 12 weeks. (n = 4) Two tailed unpaired Student's *t*‐test. (C, D) Caspase‐6 activity in eWAT (C) and iWAT (D) of C57BL/6J mice fed chow diet or HFD for 12 weeks. (n = 8) Two tailed unpaired Student's *t*‐test. (E) *CASP6* expression in the subcutaneous adipose tissue of non‐obese and obese human subjects (GSE59034). (n = 16) Two tailed unpaired Student's *t*‐test. (F‐Q) WT and C6KO mice fed HFD for 12 weeks. (F) body weight (n = 10). (G) fat mass (n = 6‐8). (H) lean mass (n = 6‐8). (I‐L) Tissue weights (n = 8‐9): eWAT (I), iWAT (J), BAT (K), and liver (L). M‐N. Lipid levels (n = 10‐18): serum triglycerides (M), and liver triglycerides (N). Data show Mean ± SEM. Two tailed unpaired Student's *t*‐test. (O) H&E staining of eWAT, iWAT, and BAT. (P, Q) Indirect calorimetry (n = 6): oxygen consumption rate (VO_2_) (P) and energy expenditure (Q). ANCOVA analysis with body weight as a covariate. *p < 0.05, **p < 0.01, ***p < 0.001, ****p < 0.0001.

Our previous study suggested that caspase‐6 contributes to the pathogenesis of metabolic disorders [3]. Other studies have identified upregulated expression of several caspase family members, including caspase‐3, ‐7, and ‐9, in obese individuals [34, 35, 36]. However, the expression profile of caspase‐6 in the context of obesity remained unclear. To address this question, we utilized a high fat diet (HFD)‐induced obesity mouse model to assess caspase‐6 levels. Western blot analysis revealed a significant upregulation of caspase‐6 in inguinal white adipose tissue (iWAT), epididymal white adipose tissue (eWAT), and brown adipose tissue (BAT) (Figure 1A,B; Figure S1A). Additionally, caspase‐6 activity was measured in eWAT and iWAT of chow diet and HFD‐fed mice. The results revealed a significant increase of caspase‐6 activity by HFD feeding (Figure 1C,D). Previous study from our group identified AMPK‐mediated phosphorylation of caspase‐6, which inhibits caspase‐6 activation [3]. As AMPK activity is repressed by HFD feeding, we assessed if phosphorylation of caspase‐6 at Ser^257^ is also reduced in HFD‐fed mice. Indeed, the results showed that HFD feeding significantly reduced caspase‐6 Ser^257^ phosphorylation in both eWAT and iWAT (Figure S1B,C). These results reaffirm our observation that HFD feeding promotes that activation of caspase‐6. To evaluate the relevance of our observation in mice to humans, we analyzed a human datasets (GSE59034) of adipose tissue from non‐obese (n = 16, age(y): 45 ± 11; BMI(kg/m^2^): 25.2 ± 2.5; Glucose(mmol/L): 5.1 ± 0.4; insulin(mU/L): 4.6 ± 2.3) and obese (n = 16, age(y): 46 ± 11; BMI(kg/m^2^): 41.4 ± 4.4; Glucose(mmol/L): 5.7 ± 1.2; insulin(mU/L): 16.0 ± 10.3) human subjects [37]. The result showed a significantly increased *CASP6* expression in obesity (Figure 1E). These findings underscore the association of adipose caspase‐6 with obesity.

### 2.2 Caspase‐6 Knockout Attenuates Diet‐Induced Obesity

To investigate whether caspase‐6 contributes to HFD‐induced obesity, we generated caspase‐6 knockout (C6KO) mice. Knockout of caspase‐6 was confirmed in iWAT, eWAT, BAT, and liver (Figure S1D). Consistent with previous report [38], C6KO mice did not exhibit any developmental defect. Under normal chow diet, caspase‐6 knockout did not affect body weight or adipose tissue mass (Figure S1E‐H). Indirect calorimetry studies showed no differences in oxygen consumption rate, carbon dioxide production, energy expenditure, respiratory exchange ratio (RER), food intake, or activity between wildtype (WT) and C6KO mice (Figure S1I‐N). However, when fed a HFD, C6KO mice exhibited significantly lower body weight compared to the WT mice (Figure 1F). Analysis of body composition by EchoMRI showed that C6KO mice had significantly less fat mass, with no difference in lean mass compared to WT mice (Figure 1G,H). Tissue weight measurements confirmed that caspase‐6 knockout significantly reduced eWAT, iWAT, BAT, and liver weight (Figure 1I–L). These findings suggest a potential role for caspase‐6 in regulating fat accumulation and overall adiposity in diet‐induced obesity. Given that reductions in fat mass are often associated with improved lipid metabolism, we assessed circulating triglyceride levels and found a significant reduction in C6KO mice (Figure 1M). Liver triglyceride levels were also significantly reduced in C6KO mice (Figure 1N). Histological analysis of adipose tissue identified smaller adipocyte size in eWAT, iWAT, and BAT of HFD‐fed C6KO mice compared to HFD‐fed WT controls (Figure 1O). Indirect calorimetry showed that C6KO significantly increased oxygen consumption rate and energy expenditure in HFD‐fed mice, without affecting food intake or activity (Figure 1P,Q; Figure S2A‐C). As these mice were fed a HFD, the increase of lipid utilization in C6KO mice did not further reduce RER (Figure S2D). Overall, these results revealed that caspase‐6 knockout increased lipolysis and energy expenditure to attenuate obesity in HFD‐fed mice.

### 2.3 Caspase‐6 Deficiency Protects Against HFD‐Induced Insulin Resistance and Inflammation

**FIGURE 2:**
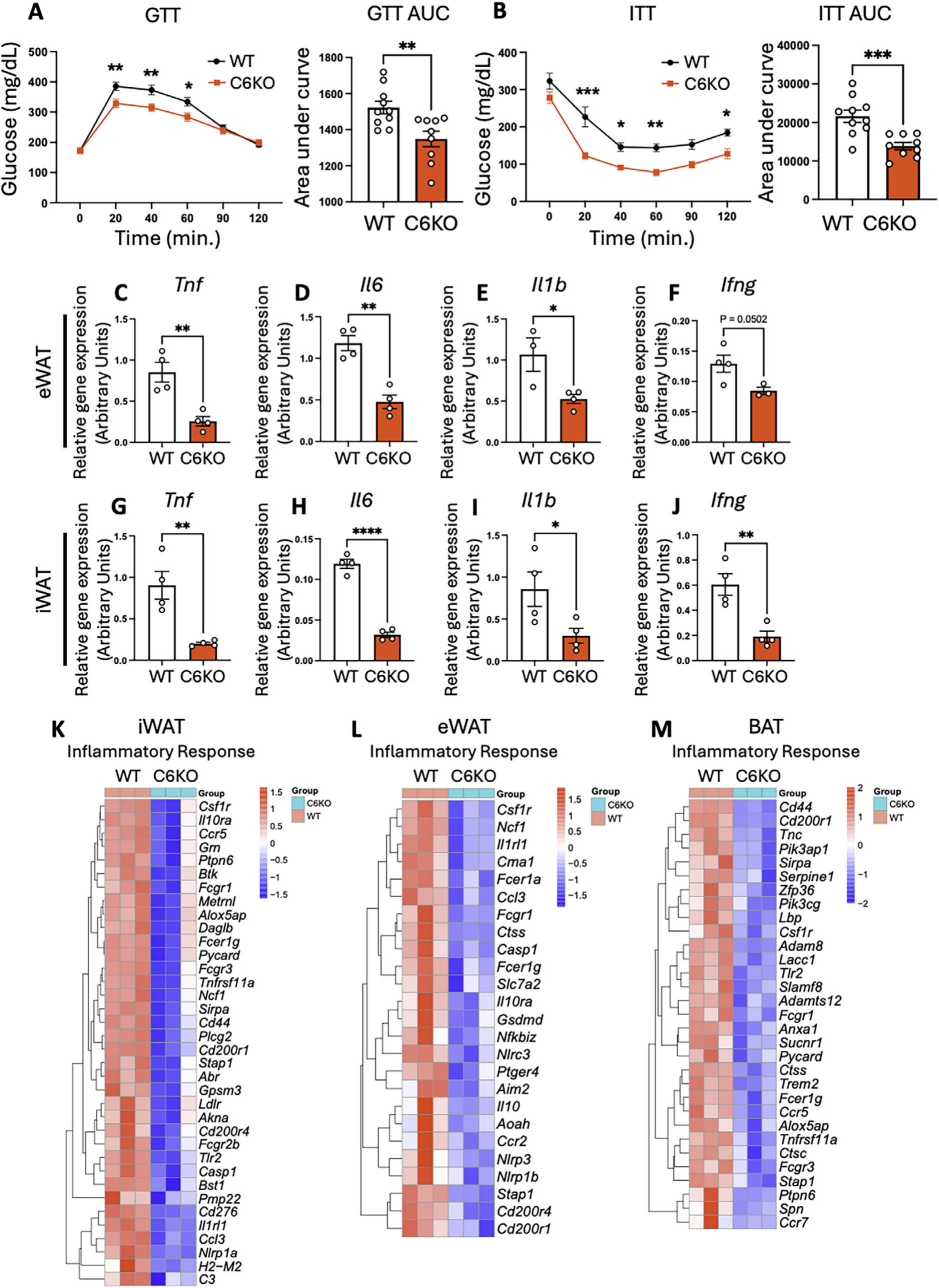
Caspase‐6 deficiency protects against HFD‐induced insulin resistance and inflammation. WT and C6KO mice fed 60% HFD for 12 weeks (A‐M). (A) Glucose tolerance test (GTT) and AUC quantification (n = 9‐10). (B) Insulin tolerance test (ITT) and AUC quantification (n = 9‐10). GTT/ITT: two‐way ANOVA followed by Šídák's‐corrected *post hoc* test. (C‐F) Expression of *Tnf* (C), *Il6* (D), *Il1b* (E), and *Ifng* (F) in eWAT. (G‐J) Expression of *Tnf* (G), *Il6* (H), *Il1b* (I), and *Ifng* (J) in iWAT. (n = 4) Two tailed unpaired Student's *t*‐test. *p < 0.05, **p < 0.01, ***p < 0.001, ****p < 0.0001. (K‐M) Relative expression values (Z‐scaled log2 [TPM + 1]) for inflammatory response genes in iWAT (K), eWAT (L), and BAT (M) from RNA‐seq (n = 3).

The attenuation of diet‐induced obesity is frequently associated with an improvement in glucose metabolism [39]. We thus examined the impact of caspase‐6 deletion on glucose metabolism in both chow diet‐fed mice and HFD‐fed mice. Fasting glucose levels, glucose tolerance test (GTT) and insulin tolerance test (ITT) showed no differences between WT and C6KO mice under chow diet feeding (Figure S3A‐C). However, when fed HFD, C6KO mice exhibited significantly lower glucose levels during both GTT and ITT, indicating an improvement of glucose tolerance and insulin sensitivity (Figure 2A,B). Notably, despite improved glucose tolerance and insulin sensitivity, there were no significant differences in basal fasting glucose (Figure 2A) or serum insulin levels (Figure S3D) between WT and C6KO mice. These findings indicate that caspase‐6 knockout protects against HFD‐induced insulin resistance and glucose intolerance.

Obesity often results in a chronic low‐grade inflammation that impairs adipose tissue functions [8, 40]. The persistent low‐grade inflammation in adipose and other metabolic tissues disrupts insulin signaling and exacerbates metabolic dysfunction [8, 40]. To better understand the role of caspase‐6 in adipose inflammation, we examined the expression of inflammatory genes in adipose tissues from HFD‐fed WT and C6KO mice. The results revealed a significant reduction in inflammatory markers, including tumor necrosis factor‐α (*Tnf*), interleukin‐6 (*Il6*), interleukin‐1β (*Il1b*), and interferon‐gamma (*Ifng*), in both eWAT and iWAT of C6KO mice compared to WT mice (Figure 2C–J). To gain deeper insight into the transcriptomic changes in adipose tissues, we performed RNA sequencing to profile the transcriptomes of eWAT, iWAT, and BAT from HFD‐fed WT and C6KO mice. PCA analysis showed that C6KO altered the transcriptomic profile of all fat depots (Figure S3E‐G). Consistent with the qPCR results, transcriptomic analysis revealed a broad downregulation of inflammatory response genes in all three fat depots of C6KO mice (Figure 2K‐M). Both Gene Ontology Biological Process (GO BP) and KEGG pathway analyses of significantly downregulated genes confirmed robust enrichment of proinflammatory pathways in C6KO adipose tissues (Figures S4–S6). Specifically, compared to WT mice, iWAT from HFD‐fed C6KO mice showed a significant downregulation of genes involved in cell adhesion, chemokine signaling, cytokine‐cytokine receptor interaction, and NF‐κB signaling pathways (Figure S4B‐G). Similarly, eWAT showed decreased expression of genes involved in cytokine‐cytokine receptor interaction, chemokine signaling pathway, cell adhesion molecules, and TLR signaling (Figure S5B‐G). In BAT, genes involved in chemokine signaling, NF‐κB signaling, and cytokine‐cytokine receptor interaction were also downregulated (Figure S6B‐F). Differentially expressed gene lists in C6KO mice are provided as Table S1 (iWAT), Table S2 (eWAT) and Table S3 (BAT). These findings indicate that C6KO mitigates adipose tissue inflammation during HFD‐induced obesity. Together, these results suggest that caspase‐6 plays a key role in regulating systemic insulin sensitivity and inflammatory responses. C6KO alleviates the activation of inflammatory pathways, thereby preserving insulin signaling and improving metabolic dysfunction during obesity.

### 2.4 Caspase‐6 Deletion Upregulates ATGL and Alters Systemic Lipid Homeostasis

**FIGURE 3:**
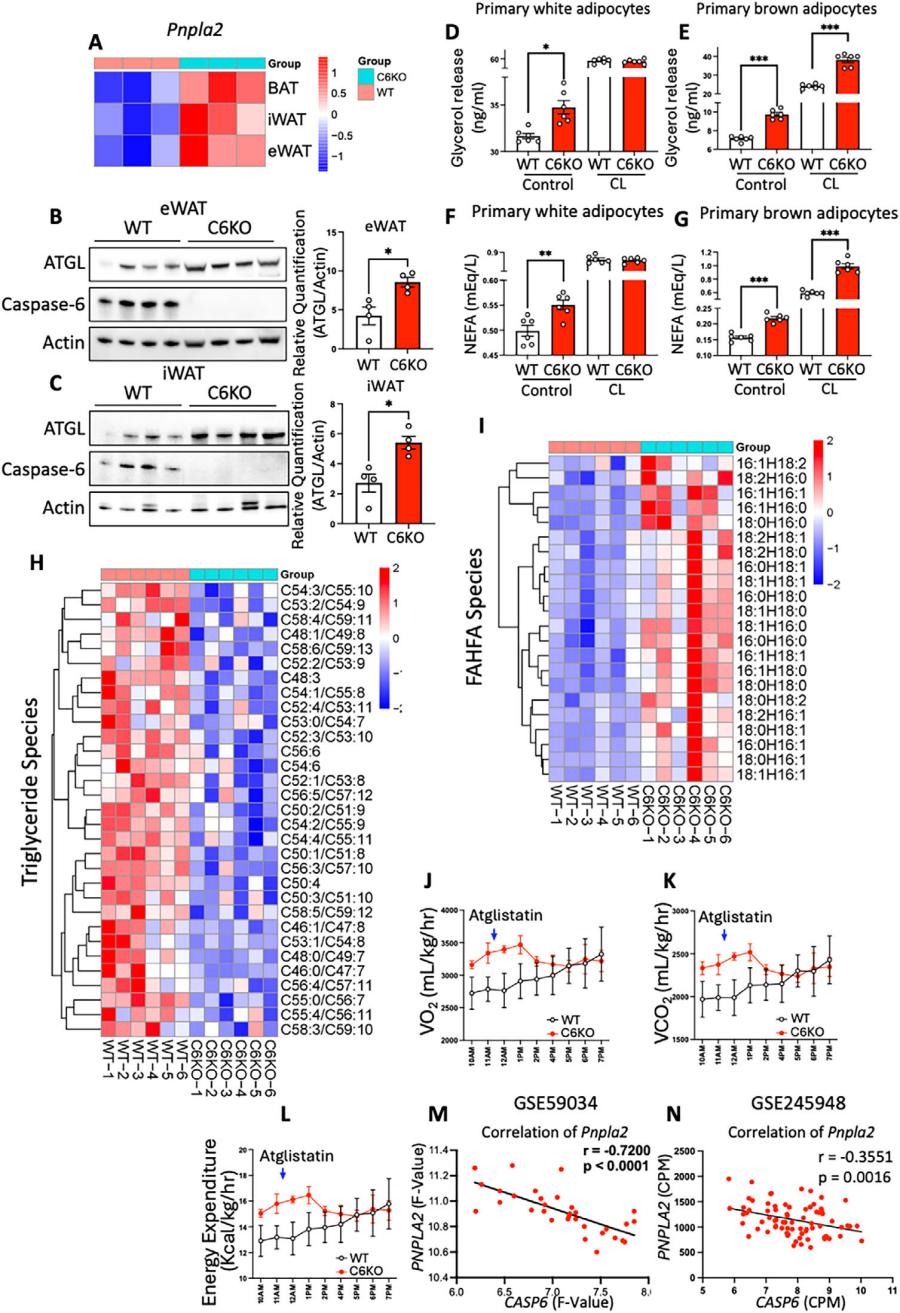
Caspase‐6 deletion upregulates ATGL and alters systemic lipid metabolism. WT and C6KO mice fed 60% HFD for 12 weeks. (A) Relative expression values (Z‐scaled log2 [TPM+1]) of ATGL (*Pnpla2*) in eWAT, iWAT, and BAT from RNA‐seq (n = 3). (B, C) Western blot and quantification of ATGL and caspase‐6 in eWAT (B) and iWAT (C). (n = 4) Two tailed unpaired Student's *t*‐test. (D‐G) Glycerol and NEFA release from white and brown adipocytes with/without CL‐316243 treatment (n = 6). Two tailed unpaired Student's *t*‐test. (H, I) Lipidomic analysis of triglyceride (H) and FAHFA (I) species in eWAT (n = 6). For each species: two tailed unpaired Student's *t*‐test. (J‐L) Indirect calorimetry on mice i.p. injected 800 µmol/kg Atglistatin (n = 3). Oxygen consumption rate (J), Carbon dioxide production (K), and energy expenditure (L). ANCOVA analysis with body weight as a covariate. (M, N) Pearson correlation between expression of *CASP6* and ATGL (*PNPLA2*) in human adipose tissue GSE59034 (M, n = 32) and GSE245948 (N, n = 76). *p < 0.05, **p < 0.01, ***p < 0.001, ****p < 0.0001.

As C6KO reduced adiposity and attenuated diet‐induced obesity, we analyzed the expression of genes involved in lipid metabolism and found a consistent upregulation of *Pnpla2* (gene symbol of ATGL) in different adipose depots of C6KO mice (Figure 3A). Western blot analysis confirmed significantly elevated ATGL protein levels in both eWAT and iWAT of C6KO mice compared to HFD‐fed WT mice (Figure 3B,C). Previous studies showed that HFD feeding reduce ATGL levels in adipose tissue [41]. Therefore, our results suggest that C6KO restores ATGL expression and improves lipid metabolism in HFD‐induced obesity. To further examine the role of caspase‐6 in regulating lipolysis, we isolated primary preadipocytes from white and brown adipose tissues of WT and C6KO mice. These preadipocytes were differentiated and treated with the β3‐adrenergic receptor agonist CL‐316,243 to stimulate lipolysis. Measurements of free glycerol and non‐esterified fatty acids (NEFAs) in the culture medium showed enhanced basal lipolysis in C6KO adipocytes, as evidenced by increased release of free glycerol and non‐esterified fatty acids (NEFAs) (Figure 3D–G). Interestingly, CL‐316,243‐stimulated lipolysis was increased in brown adipocytes from C6KO mice, but not in white adipocytes. Since CL‐316,243 activates the cyclic adenosine monophosphate (cAMP)‐protein kinase A (PKA) pathway, which in turn activates hormone sensitive lipase (HSL) to hydrolyze diacylglycerol (DAG) into fatty acids and monoacylglycerol (MAG), this observation suggests that enhanced lipolysis in brown adipocytes might depend on ATGL‐mediated DAG production, which provides the substrate for HSL to hydrolyze the second fatty acid. To further validate these findings, we performed in vivo lipolysis study, in which we injected WT and C6KO mice on chow diet or HFD with either PBS or 0.5 mg/kg CL‐316,243. CL‐316,243 induced lipolysis to increase serum glycerol and NEFAs in both chow diet and HFD‐fed mice. Interestingly, the results showed that C6KO increased basal levels of serum glycerol and NEFAs, and further enhanced CL‐316,243‐induced release of glycerol and NEFAs in HFD‐fed mice, but not in chow diet‐fed mice (Figure S7A).

To validate the increase of ATGL activity in vivo, we performed lipidomics analysis on adipose tissues from HFD‐fed WT and C6KO mice. C6KO significantly reduced the levels of most triglyceride species (Figure 3H; Figure S7B‐F and Table S4). Notably, a previous study identified ATGL as a biosynthetic enzyme responsible for the production of FAHFAs, a class of lipids exhibiting metabolically beneficial effects. They were shown to attenuate inflammation and improve glucose metabolism via reducing proinflammatory cytokine production and enhancing GLP‐1 secretion [13, 14, 15, 16, 17, 18]. This connection between ATGL activity and FAHFAs highlights the potential role of caspase‐6 in modulating FAHFA levels through its regulation of ATGL. To investigate whether C6KO affects ATGL activity to regulate FAHFAs, we quantified FAHFA species in white adipose tissue from HFD‐fed WT and C6KO mice. The results showed a significant increase of FAHFAs in C6KO adipose tissue, further supporting enhanced ATGL activity in C6KO mice (Figure 3I; Figure S7G,H and Table S4). This increase of FAHFA may contribute to reduced inflammation and improved insulin sensitivity in HFD‐fed C6KO mice. Together, these data provide compelling evidence that caspase‐6 is a critical regulator of ATGL levels in adipose tissue.

To determine whether increased ATGL‐mediated lipolysis is responsible for the elevated energy expenditure in C6KO mice, we treated HFD‐fed WT and C6KO mice with Atglistatin, a selective ATGL inhibitor, and assessed energy expenditure by indirect calorimetry. ATGL inhibition reduced oxygen consumption rate and energy expenditure in C6KO mice and completely diminished the metabolic difference between C6KO mice and WT mice (Figure 3J–L; Figure S7I‐L). The results confirmed that the increased ATGL activity drives the elevated energy expenditure in C6KO mice during HFD feeding.

Our studies utilizing a transgenic mouse model suggest that caspase‐6 is a key regulator of ATGL activity in diet‐induced obesity. To evaluate the relevance of our findings in humans, we analyzed two independent human transcriptomic datasets. The first dataset (GSE59034) analyzes subcutaneous adipose tissues from non‐obese (n = 16, age(y): 45 ± 11; BMI(kg/m^2^): 25.2 ± 2.5; Glucose(mmol/L): 5.1 ± 0.4; insulin(mU/L): 4.6 ± 2.3) and obese (n = 16, age(y): 46 ± 11; BMI(kg/m^2^): 41.4 ± 4.4; Glucose(mmol/L): 5.7 ± 1.2; insulin(mU/L): 16.0 ± 10.3) human subjects [37]. Transcriptomic analysis revealed a significant negative correlation between *CASP6* and *PNPLA2* (ATGL) expression (Figure 3M), suggesting that lower *CASP6* expression is associated with higher *PNPLA2* expression. A second dataset (GSE245948), which included subcutaneous adipose tissue samples from 76 individuals (age(y): 52 (44‐57); BMI(kg/m^2^): 38.6 (34.6‐43.5); HOMA‐IR: 4.4 (3.1‐7.7)) with obesity and prediabetes [42], confirmed this negative correlation (Figure 3N). Collectively, these findings reinforce the conclusion that caspase‐6 negatively regulates ATGL levels in adipose tissues, with implications for both murine and human metabolic health.

### 2.5 Caspase‐6 Regulates Mitochondrial Function in Diet‐Induced Obesity

**FIGURE 4:**
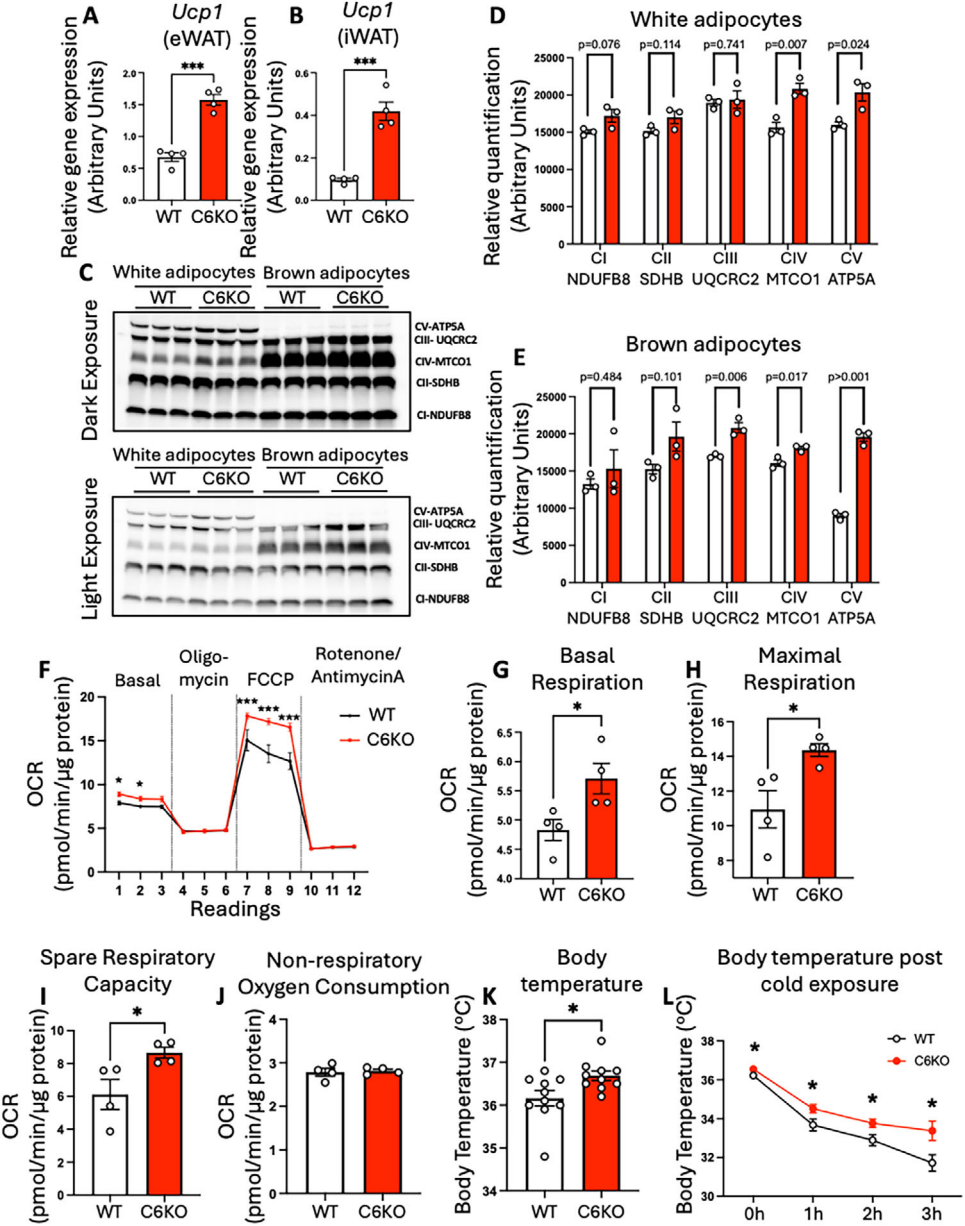
Caspase‐6 regulates mitochondrial function in diet‐induced obesity. WT and C6KO mice fed 60% HFD for 12 weeks. (A, B) *Ucp1* mRNA levels in eWAT (A) and iWAT (B) (n = 4). Two tailed unpaired Student's *t*‐test. (C) Immunoblot of OXPHOS complexes I‐V in white and brown adipocytes. (D, E) Quantification of mitochondrial complexes (n = 3). Two tailed unpaired Student's *t*‐test. (F) Seahorse assay on primary white adipocytes (n = 4). Two‐way ANOVA followed by Šídák's‐corrected *post hoc* test. (G‐J) Basal (G), maximal (H), spare respiratory capacity (I), and non‐respiratory oxygen consumption (J) from Seahorse assay (n = 4). Two tailed unpaired Student's *t*‐test. (K, L) Rectal temperature at ambient temperature (K) and after 4°C cold exposure (L) (n = 8‐10). Two tailed unpaired Student's *t*‐test for K; two‐way ANOVA followed by Šídák's‐corrected *post hoc* test for L. *p < 0.05, **p < 0.01, ***p < 0.001, ****p < 0.0001.

As C6KO increased lipolysis and energy expenditure in HFD‐fed mice, we examined if C6KO also affects mitochondrial function and thermogenesis. Gene expression analysis by qPCR showed that C6KO significantly upregulated *Ucp1* expression in white adipose tissues (Figure 4A,B), suggesting an elevation of thermogenesis in adipose tissues. To determine whether caspase‐6 affects mitochondria biogenesis and function, we examined the mRNA and protein levels of different complexes of mitochondrial respiratory chain (MRC). Primary mature adipocytes were isolated from white and brown adipose tissues of HFD‐fed WT and C6KO mice. Western blot analysis revealed that C6KO moderately increased MRC proteins, with significant upregulation of complex IV and V in white adipocytes (Figure 4C,D), and complex III, IV, and V in brown adipocytes (Figure 4C,E). QPCR analysis further revealed that C6KO upregulated expression of key MRC and mitochondrial biogenesis genes, including *Cox4i1* and *Ndufv1* in eWAT, *Atp5f1b*, *Ndufv1*, and *Ppargc1a* in iWAT (Figure S8A‐J). To assess whether this moderate increase in mitochondrial content translates to improved mitochondrial function, we performed Seahorse assays on differentiated primary preadipocytes isolated from white adipose tissue of WT and C6KO mice. C6KO adipocytes exhibited significantly enhanced basal respiration, maximal respiration, and spare respiratory capacity, with no change in non‐respiratory oxygen consumption when electron transport chain is completely inhibited (Figure 4F–J). These findings suggest that caspase‐6 deletion improves mitochondrial function in adipocytes, potentially contributing reduced adiposity in HFD‐fed mice.

To validate if improved mitochondrial function by C6KO enhances thermogenesis in vivo, we measured rectal temperatures in HFD‐fed WT and C6KO mice. C6KO mice exhibited significantly higher basal body temperature, indicating enhanced thermogenesis (Figure 4K). Additionally, during a cold tolerance test, C6KO mice maintained higher body temperatures compared to WT mice, further supporting enhanced thermogenic capability (Figure 4L). Indirect calorimetry also showed that HFD‐fed C6KO mice exhibited markedly increased oxygen consumption rate and energy expenditure during cold exposure (Figure S8K‐M), supporting that caspase‐6 deficiency enhanced cold‐induced thermogenesis. In contrast, the thermoneutral condition (30°C) that represses thermogenesis diminished the difference in oxygen consumption rate and energy expenditure in WT and C6KO mice (Figure S8N‐P). Collectively, these studies suggest that caspase‐6 deficiency enhances mitochondrial function and thermogenesis, contributing to improved metabolic outcomes in the context of HFD‐induced obesity.

### 2.6 Caspase‐6 Cleaves PPARγ and SP1 to Control ATGL Expression in Adipocytes

**FIGURE 5:**
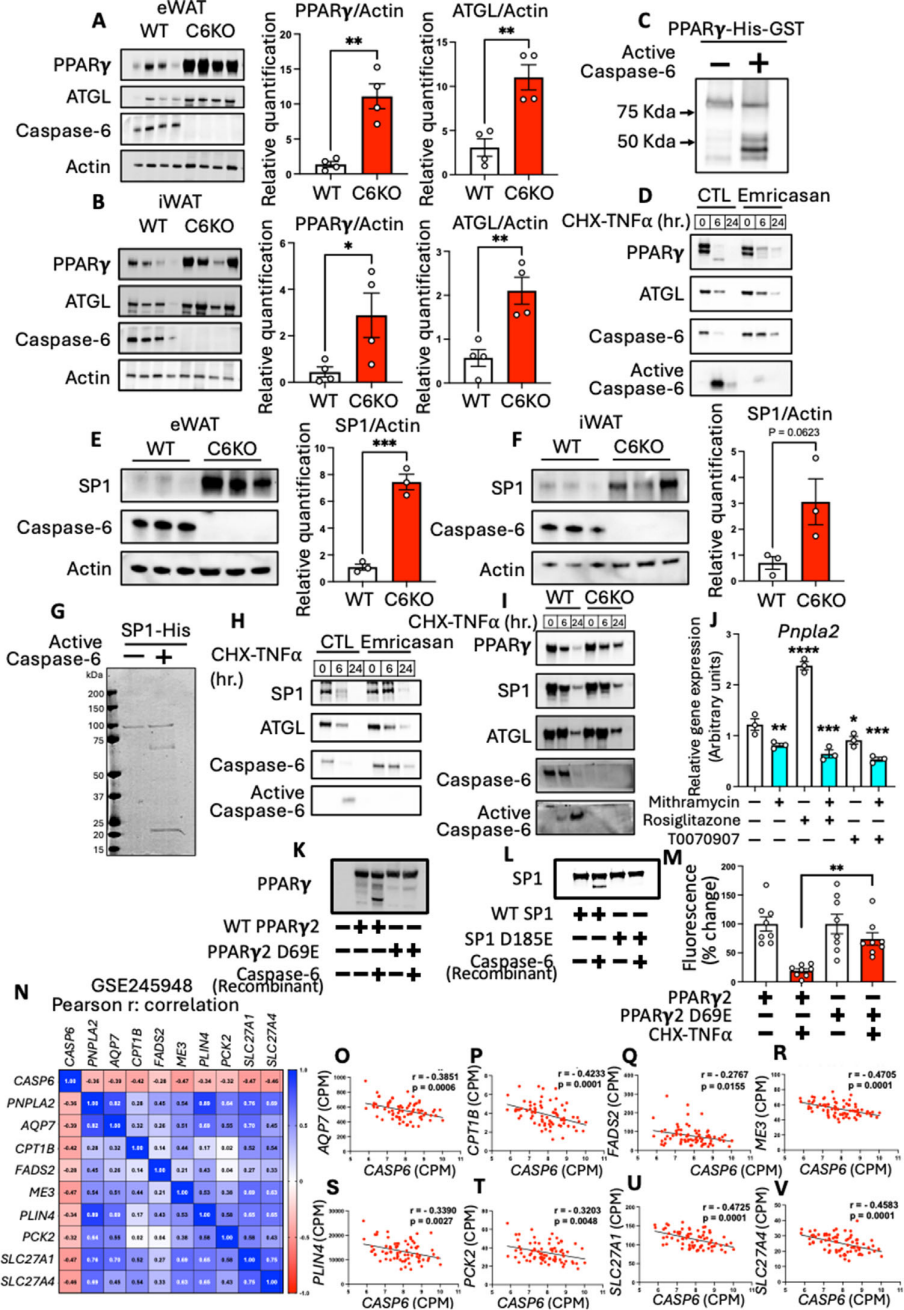
Caspase‐6 cleaves PPARγ and SP1 to control ATGL expression in adipocytes. (A, B) Immunoblot of PPARγ and ATGL proteins in eWAT (A) and iWAT (B) of WT and C6KO mice fed 60% HFD for 12 weeks (n = 4). Two tailed unpaired Student's *t*‐test. (C) In vitro cleavage of recombinant PPARγ by active caspase‐6. Immunoblot of PPARγ. (D) Immunoblot of lysates from 3T3‐L1 adipocytes treated with cycloheximide (5 µg/ml) and TNFα (25 ng/ml) for indicated time with or without pretreatment of Emricasan (50 µg/ml) for 1 hr. (E, F) Immunoblot SP1 protein in eWAT (E) and iWAT (F) of WT and C6KO mice fed HFD for 12 weeks (n = 3). Two tailed unpaired Student's *t*‐test. (G) In vitro cleavage of recombinant SP1 by active caspase‐6. Coomassie blue staining of gel. (H) Immunoblot of lysates from 3T3‐L1 adipocytes treated with cycloheximide (CHX, 5 µg/ml) and TNFα (25 ng/ml) for indicated time with or without pretreatment of Emricasan (50 µg/ml) for 1 hr. (I) Immunoblot of lysates from ex vivo cultured eWAT from WT or C6KO, treated with CHX (15 µg/ml) and TNFα (75 ng/ml) for indicated time. (J) Pnpla2 expression in 3T3‐L1 adipocytes treated with Rosiglitazone (5µM) or T0070907 (100 nM) in the presence or absence of Mithramycin (10 µM) for 6 h (n = 3). Two tailed unpaired Student's *t*‐test. (K) In vitro cleavage of WT and mutant (D69E) PPARγ2 expressed in HEK293T by recombinant active caspase‐6. (L) In vitro cleavage of WT and mutant (D185E) SP1 expressed in HEK293T by recombinant active caspase‐6. (M) PPARγ activity reporter assay in HEK293T cell expressing PPRE‐H2B‐eGFP along with WT PPARγ or PPARγ D69E mutant in the absence or presence of CHX‐TNFα treatment (n = 8). Two tailed unpaired Student's *t*‐test. (N‐V) Pearson correlation between *CASP6* and PPARγ target genes in human adipose tissues (GSE245948): Correlation matrix (N), *AQP7* (O), *CPT1B* (P), *FADS2* (Q), *ME3* (R), *PLIN4* (S), *PCK2* (T), *SLC27A1* (U), and *SLC27A4* (V). (n = 76). *p < 0.05, **p < 0.01, ***p < 0.001, ****p < 0.0001.

To understand how caspase‐6 regulates adipose biology, particularly ATGL expression, we performed KEGG pathway analysis of RNAseq data from WT and C6KO mice and found a consistent upregulation of PPAR signaling pathway across all three adipose depots (Figure S9A‐C). PPARγ is a critical transcriptional regulator of adipocyte function and metabolic homeostasis [21]. The cleavage of PPARγ leads to its degradation, thus reducing PPARγ protein levels in adipocytes [43]. Moreover, previous study showed that PPARγ and its cofactor SP1 coordinately control *Pnpla2* expression in adipocytes [23]. In our study, we found that HFD feeding reduces PPARγ and its cofactor SP1 in different fat depots (Figure S9D‐F). To determine if PPARγ is a substrate of caspase‐6 and mediates the regulation of ATGL, we first examined the mRNA and protein levels of PPARγ in adipose tissues of HFD‐fed WT and C6KO mice. The results showed that C6KO significantly increased both PPARγ and ATGL proteins in adipose tissues of HFD‐fed mice, without affecting PPARγ mRNA levels (Figure 5A,B; Figure S9G,H). To uncover how caspase‐6 regulates PPARγ, we performed an in vitro cleavage assay [3] using recombinant PPARγ and active caspase‐6 and found that caspase‐6 directly cleaved PPARγ, generating truncated protein fragments (Figure 5C), which could be degraded in cells and in vivo. To confirm the regulatory role of PPARγ on *Pnpla2* expression, we treated 3T3‐L1 adipocytes with T0070907 (a potent PPARγ antagonist), or Rosiglitazone (a PPARγ agonist). The results confirmed that PPARγ inhibition significantly reduced both mRNA and protein levels of ATGL (Figure S9I,J). Conversely, Rosiglitazone significantly upregulated ATGL mRNA and protein levels (Figure S9K,L). These results confirmed that PPARγ directly regulates *Pnpla2* (ATGL) expression in adipocytes. Furthermore, these results suggest that caspase‐6 regulates PPARγ protein levels to modulate the expression of PPARγ target genes, including *Pnpla2*. To investigate the role of caspase activation in PPARγ regulation, we treated 3T3‐L1 adipocytes with low dose cycloheximide (CHX) and TNFα, with or without caspase inhibitor Emricasan. Activation of caspase‐6 reduced both PPARγ and ATGL levels, while this effect was attenuated by Emricasan (Figure 5D). To further validate that caspase‐6 deficiency increases PPARγ activity in adipose tissue, we compared the effects of C6KO and PPARγ activation by analyzing the gene expression profiles in adipose tissues from WT/C6KO mice and HFD‐fed mice treated with the PPARγ agonist rosiglitazone (GSM2420483). Interestingly, this analysis revealed 47 overlapping upregulated genes including multiple canonical PPARγ target genes. Notably, *Pnpla2* (ATGL) was among the commonly upregulated genes (Figure S9M). This finding further supports the notion that caspase‐6 deficiency enhances PPARγ‐induced gene transcription that contributes to the improved metabolic phenotypes. Overall, these data provide compelling evidence that caspase‐6 directly cleaves PPARγ, thereby reducing ATGL expression and impairing lipolytic function and adipose tissue metabolism.

Functioning as a cofactor of PPARγ, SP1 also regulates the expression of *Pnpla2* [23]. Interestingly, we found that C6KO significantly increased SP1 protein levels in both eWAT and iWAT of HFD‐fed mice, without altering its mRNA levels (Figure 5E,F; Figure S9N,O). This observation led us to examine if SP1 is a direct substrate of caspase‐6. In an in vitro cleavage assay using recombinant SP1 and active caspase‐6, we observed that SP1 was cleaved into two fragments, confirming it as a caspase‐6 substrate (Figure 5G). To further investigate the role of caspase‐6 activation in SP1 regulation, we treated 3T3‐L1 cells with CHX and TNFα, in the absence or presence of Emricasan. Caspase‐6 activation reduced SP1 and ATGL levels, and the effect is attenuated by Emricasan (Figure 5H). Considering that Emricasan acts as a pan caspase inhibitor, to confirm the specific role of caspase‐6 in regulating PPARγ and SP1 proteins, we performed an *ex vivo* experiment, in which adipose tissue from WT and C6KO mice was isolated and subjected to TNFα and CHX treatment. The results showed that caspase‐6 KO attenuated TNFα and CHX‐induced reduction of PPARγ and SP1, indicating a specific role of caspase‐6 in mediating proteolytic degradation of PPARγ and SP1. (Figure 5I). These findings supported that caspase‐6 directly regulates PPARγ and SP1 levels. Furthermore, we examined if SP1 is required for PPARγ‐mediated induction of *Pnpla2* expression. 3T3‐L1 adipocytes were treated with Rosiglitazone (PPARγ agonist) or T0070907 (antagonist), with or without the SP1 inhibitor mithramycin. Mithramycin downregulated basal *Pnpla2* expression and completely abrogated its induction by Rosiglitazone. Although T0070907 alone already reduced the expression of *Pnpla2*, co‐treatment with mithramycin produced an additive inhibitory effect (Figure 5J). These findings supported that PPARγ and SP1 act synergistically to regulate *Pnpla2* expression in adipocytes. Together, these data indicate that caspase‐6 cleaves PPARγ and SP1 to modulate the expression of ATGL in adipocytes.

PPARγ1 and PPARγ2 are alternative transcription and splicing products of *Pparg* gene. PPARγ2 contains 30 additional amino acid at the N‐terminus, while the remaining sequence is completely identical between PPARγ1 and PPARγ2, including all potential caspase‐6 substrate motif. Previous studies showed that PPARγ2 is predominantly expressed in adipocytes and plays a critical in metabolic regulation [44, 45]. To further understand caspase‐6‐mediated cleavage of PPARγ and SP1, we performed in silico analysis to identify caspase‐6 cleavage sites by comparing amino acid sequences of PPARγ2 and SP1 with caspase‐6 substrate motif. The analysis identified 5 potential caspase‐6 cleavage sites on PPARγ2 and 2 potential sites on SP1 (Figure S9P). Next, we conducted site‐directed mutagenesis to introduce Asp(D) to Glu(E) mutation at the potential caspase‐6 cleavage sites in PPARγ2 and SP1. The D to E mutation makes the substrate uncleavable while maintaining the negatively charged side chain. Wildtype (WT) and mutants of PPARγ and SP1 were expressed in HEK293T cells. Cell lysates were used for in vitro cleavage assay by recombinant active caspase‐6. The results revealed that caspase‐6 cleaves PPARγ2 at Asp^69^ (equivalent to PPARγ1 Asp^39^) and SP1 at Asp^185^ (Figure 5K,L). To further validate the impact of caspase‐6‐mediated cleavage on PPARγ activity, we adopted a PPARγ activity reporter assay using HEK293T cells expressing the PPRE(PPARγ response element)‐H2B‐eGFP (addgene #84393) [46]. These cells were transfected with either WT PPARγ2 or the non‐cleavable PPARγ2 D69E mutant. Following transfection, cells were subjected to CHX‐TNFα treatment for 8 h, and GFP fluorescence intensity was quantified (Figure 5M). After CHX‐TNFα treatment, cells expressing WT PPARγ2 exhibited a marked reduction in GFP fluorescence, indicating diminished PPARγ activity upon cleavage. In contrast, cells expressing the PPARγ2 D69E mutant were able to maintain fluorescence intensity even after CHX‐TNFα treatment. This observation confirms that the D69E mutation prevents caspase‐6‐mediated PPARγ cleavage and subsequent loss of PPARγ activity. These results provide strong evidence that caspase‐6 cleaves PPARγ2 Asp^69^ to regulate its activity.

To uncover the fate of cleaved PPARγ fragment, we pretreated 3T3‐L1 adipocytes with the proteasome inhibitor MG132 to block proteasomal degradation, then treated them with CHX‐TNFα. Inhibition of proteasome resulted in the stabilization and accumulation of cleaved PPARγ fragment. Interestingly, MG132 also attenuated the cleavage of PPARγ, possibly due to the accumulation of truncated PPARγ fragment (Figure S9Q). These findings indicate that the cleaved product is targeted for proteasomal degradation. Moreover, to further confirm the role of caspase‐6 in the cleavage of PPARγ and SP1, as well as its impact on ATGL expression, we overexpressed Myc‐caspase‐6 in 3T3‐L1 adipocytes. Upon CHX‐TNFα treatment, adipocytes overexpressing caspase‐6 showed enhanced cleavage and loss of both PPARγ and SP1, accompanied by a concomitant reduction in ATGL levels (Figure S9R). Taken together, our data support that caspase‐6 cleaves both PPARγ and SP1 to inhibit *Pnpla2* expression in adipocytes. This regulation underscores a novel mechanism by which proteolytic regulation intersects with transcriptional control in adipose tissue.

To determine the human relevance of this mechanism, we analyzed those two independent transcriptomic datasets from human adipose tissues. In the first dataset (GSE245948), which includes subcutaneous adipose tissue samples from 76 individuals with obesity and prediabetes, correlation analysis revealed a strong negative association between *CASP6* and multiple PPARγ target genes, as illustrated in the correlation matrix (Figure 5N). Further statistical analysis confirmed significantly negative correlations between caspase‐6 and PPARγ target genes, such as *AQP7, ME3, PLIN4, etc* (Figure 5O–V). Consistent results were observed in the second dataset (GSE59034), which includes subcutaneous adipose tissue of non‐obese and obese patients. The analysis of this dataset confirmed a strong negative correlation between *CASP6* and PPARγ target genes (Figure S9S‐AA). Collectively, these results support our hypothesis that caspase‐6 negatively regulates PPARγ signaling in adipose tissue, thereby downregulating ATGL levels.

### 2.7 Adipocyte‐Specific Caspase‐6 Knockout Protects Against Diet‐Induced Obesity and Insulin Resistance

**FIGURE 6:**
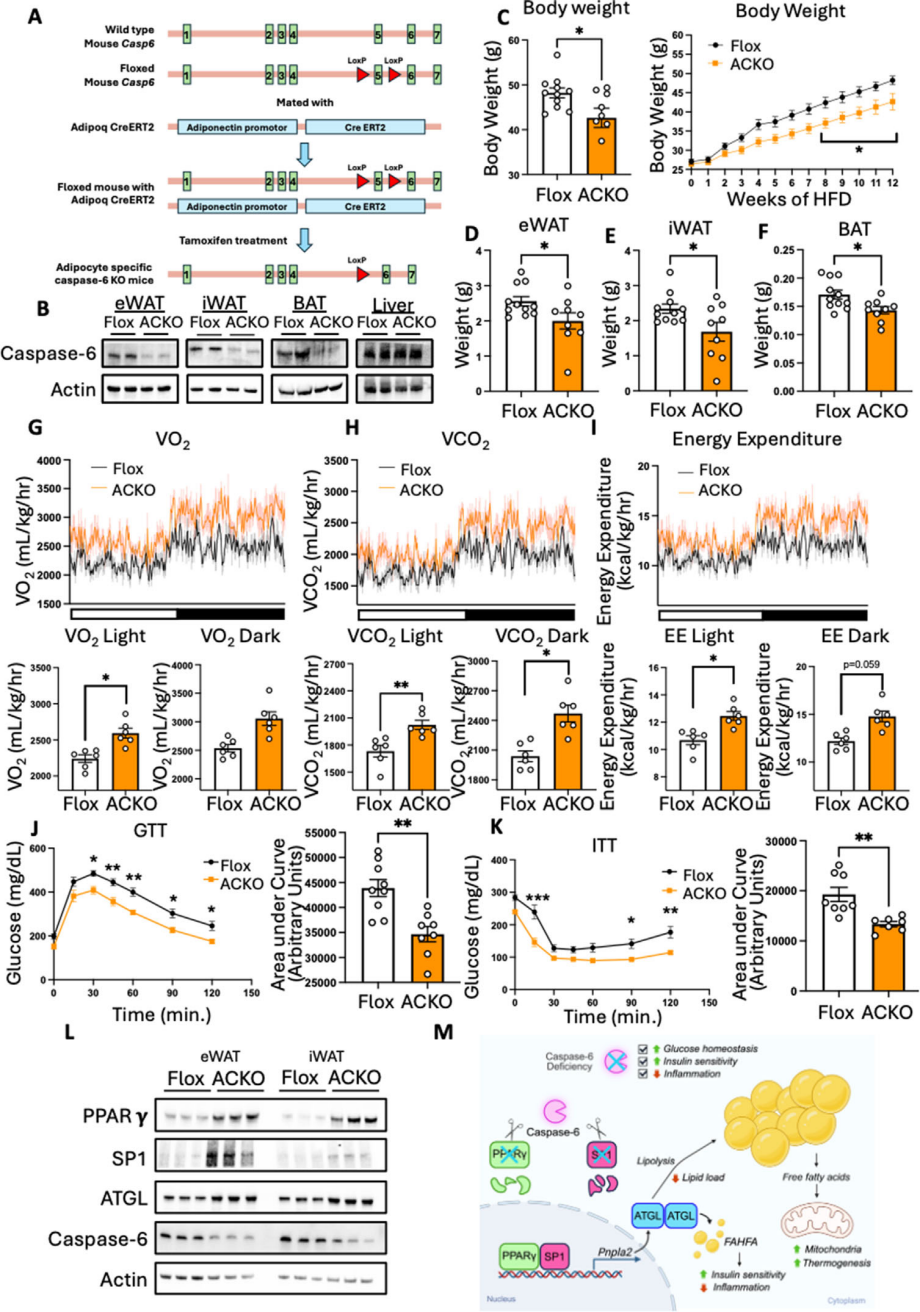
Adipocyte‐specific caspase‐6 knockout protects against diet‐induced obesity and insulin resistance. Flox and adipocyte‐specific caspase‐6 knockout (ACKO) mice fed 60% HFD for 12 weeks. (A) Schematic diagram of generating Casp6 flox (Flox) mice and adipocyte‐specific Casp6 knockout (ACKO) mice. (B) Immunoblot of caspase‐6 protein in adipose tissues and livers. (C) Body weight (n = 9‐11). (D‐F) Tissue weights (n = 9‐11): eWAT (D), iWAT (E), BAT (F). Two tailed unpaired Student's *t*‐test. (G–I) Indirect calorimetry (n = 6): oxygen consumption rate (G), carbon dioxide production (H), and energy expenditure (I). ANCOVA analysis with body weight as a covariate. (J, K) GTT (J, n = 9‐10) and ITT (K, n = 8‐9) with AUC quantification. GTT/ITT: two‐way ANOVA followed by Šídák's‐corrected *post hoc* test. (L) Immunoblots for the expression levels of PPARγ, SP1 and ATGL in eWAT and iWAT of Flox and ACKO mice fed HFD for 12 weeks. (M) Graphical summary. *p < 0.05, **p < 0.01, ***p < 0.001, ****p < 0.0001.

In this study, we have shown that global caspase‐6 knockout attenuates HFD‐induced obesity and insulin resistance. To confirm that these protective effects attribute to the loss of caspase‐6 in adipocytes, we generated adipocyte‐specific caspase‐6 knockout (ACKO) mice. Caspase‐6 flox (Flox) mice were created by flanking Exon 5 of the *Casp6* gene with LoxP sites and were then crossed with adiponectin‐Cre^ERT2^ mice, which express tamoxifen‐inducible Cre recombinase under the control of the adiponectin promoter (Figure 6A). Following tamoxifen injection, we confirmed successful deletion of caspase‐6 in eWAT, iWAT, and BAT, but not in the liver (Figure 6B). Under chow diet condition, ACKO and Flox mice showed comparable body weights and adipose tissue masses (eWAT, iWAT, BAT) (Figure S10A‐D). Fasting glucose levels, GTT and ITT showed no differences in glucose metabolism or insulin sensitivity between the two groups (Figure S10E‐G). Indirect calorimetry analysis also showed no significant differences in oxygen consumption rate, energy expenditure, respiratory exchange rate, food intake, or physical activity (Figure S10H‐M). These findings suggest that adipocyte‐specific deletion of caspase‐6 does not alter glucose or energy metabolism under normal chow diet feeding.

However, under HFD feeding, ACKO mice exhibited significantly lower body weight and reduced eWAT, iWAT, and BAT mass compared to Flox mice (Figure 6C–F). Indirect calorimetry analysis revealed that adipocyte‐specific caspase‐6 knockout significantly increased oxygen consumption rate and energy expenditure, without altering respiratory exchange rate, food intake, or activity (Figure 6G–I; Figure S10N‐P). These data confirmed that adipocyte‐specific caspase‐6 knockout increases energy expenditure and protects against diet‐induced obesity, recapitulating the phenotype observed in whole body caspase‐6 knockout mice. Next, we examined if ACKO also improves glucose metabolism and insulin sensitivity, we performed GTT and ITT in HFD‐fed mice. While fasting blood glucose was moderately lower in ACKO mice, adipocyte‐specific caspase‐6 knockout resulted in significantly improved glucose tolerance and insulin sensitivity in HFD‐fed mice (Figure 6J,K). Furthermore, consistent with our observation in C6KO mice, PPARγ, SP1, and ATGL levels were markedly increased in both eWAT and iWAT of HFD‐fed ACKO mice, compared to the Flox mice. (Figure 6L). Taken together, our studies showed that adipocyte‐specific caspase‐6 knockout attenuates HFD‐induced obesity and insulin resistance, mirroring the effects observed in whole body caspase‐6 knockout mice. These results highlight a critical role of adipocyte caspase‐6 in modulating systemic metabolic homeostasis under conditions of HFD feeding.

## 3 Discussion

Our previous study established caspase‐6 as a mediator of hepatocellular death and liver injury in MASH [3], suggesting its involvement in metabolic diseases. In this study, we found that caspase‐6 is induced in adipose tissue during HFD‐induced obesity and plays a crucial role in regulating energy and lipid metabolism. Both whole body and adipocyte‐specific caspase‐6 knockout attenuate diet‐induced obesity by increasing lipolysis and energy expenditure. Transcriptomic analysis revealed that caspase‐6 deficiency upregulated the expression of *Pnpla2* (ATGL), a key lipase in adipose tissues. Mechanistic studies revealed that caspase‐6 directly cleaves PPARγ and SP1, two transcription factors that control *Pnpla2* expression. Lipidomic analysis confirmed increased ATGL activity in C6KO mice, evidenced by reduced triglyceride accumulation in adipose tissue. Pharmacological inhibition of ATGL with Atglistatin attenuated the metabolic benefits of caspase‐6 deficiency, validating that enhanced ATGL activity drives the observed increase in energy expenditure. In addition to its established role in triglyceride hydrolysis, ATGL was recently shown to catalyze the synthesis of FAHFAs via its transacylase activity [13, 14, 15, 16, 17, 18]. Our lipidomics study showed that FAHFA species were significantly increased in adipose tissue of C6KO mice, further explaining the beneficial metabolic phenotype associated with caspase‐6 deficiency. Collectively, our study revealed a new Casp6‐PPARγ/SP1‐ATGL axis that governs lipid mobilization and energy balance in the context of diet‐induced obesity (Figure 6M), positioning caspase‐6 as a potential therapeutic target for metabolic disorders.

ATGL is a rate limiting enzyme for the hydrolysis of triglycerides [11, 12, 47, 48]. Studies have shown that adipocyte specific overexpression of ATGL decreases adipocyte triglycerides content and attenuates diet induced obesity [11]. In contrast, ATGL deficient mice exhibit increased fat mass and ectopic deposition of triglycerides, impaired cold adaptation, and cardiac myopathy as a result of excessive lipid accumulation [49]. In humans, ATGL mutations are associated with Neutral lipid storage disease (NSLD) with myopathy [50]. Additionally, ClinVar identifies at least 55 mutations in *Pnpla2* (ATGL) to be pathogenic due to the disruption of lipid metabolism [51]. These clinical and preclinical data align with our findings, reinforcing that enhanced ATGL expression driven by caspase‐6 deficiency improves metabolic health.

The transcriptional regulation of ATGL is a complex process involving multiple factors [52, 53, 54]. Notably, transcription factors PPARγ and SP1 have been identified as key regulators [23, 54]. Systemic PPARγ deletion causes severe type 2 diabetes, characterized by hyperglycemia and hyperinsulinemia [55]. Furthermore, adipocyte‐specific knockout of PPARγ leads to insulin resistance [56]. In contrast, activation of PPARγ by thiazolidinediones enhances insulin sensitivity and protects against inflammation and obesity associated metabolic diseases [57, 58]. Human studies revealed that adipose tissue PPARγ levels were positively associated with weight loss after sleeve gastrectomy [59]. Dominant negative mutations in human PPARγ are associated with severe insulin resistance, diabetes mellitus as well as hypertension [60]. Additionally, loss‐of‐function mutations in *PPARG* result in familial partial lipodystrophy type 3 (FPLD3) and are frequently associated with profound metabolic derangements in affected individuals [61]. These findings highlighted the critical roles of PPARγ in regulating glucose and lipid metabolism. In a previous study, Roy et al. demonstrated coordinated transcriptional control of adipocyte ATGL expression by PPARγ and SP1 [23]. Our study discovered the intricate interplay between caspase‐6 and transcription factors PPARγ and SP1. We found that caspase‐6 reduces PPARγ and SP1 levels via direct proteolytic cleavage, thus suppressing *Pnpla2* expression. Previous studies reported that, in cultured 3T3‐L1, caspase‐1, ‐3, ‐6, and ‐8 could contribute to the cleavage and degradation of PPARγ [43, 62]. Here, by combining new transgenic mouse models with in vitro cleavage assay using recombinant proteins, we show that caspase‐6 plays a predominant role in the cleavage and degradation of both PPARγ and SP1, thus regulating *Pnpla2* expression, in adipose tissues of HFD‐fed mice. HFD feeding downregulates ATGL in adipose tissues [41]. Our findings further show that caspase‐6 mediates this effect via reducing both PPARγ and SP1 in adipose tissues in diet‐induced obesity. Caspase‐6 knockout restores PPARγ, SP1, and ATGL levels, which are essential for maintaining metabolic homeostasis.

While PPARγ is required for the differentiation and lipid accumulation in preadipocytes, its activation induces beiging of white adipocytes to promote energy expenditure in HFD‐fed mice [26, 27, 28, 29, 30]. In our study, we observed a moderate increase of mitochondrial content and an elevation of mitochondrial function in adipocytes and adipose tissues of caspase‐6 knockout mice during HFD feeding. Two mechanisms could lead to this effect. First, activation of PPARγ is known to induce mitochondrial biogenesis by regulating PPARγ coactivation 1 alpha (PGC1α), the key transcription factor inducing mitochondrial biogenesis [63, 64, 65]. Second, previous studies demonstrated that fatty acids, particularly long chain fatty acids, released during lipolysis activate UCP1 to induce thermogenesis [66, 67]. In our study, the increased ATGL activity enhances fatty acid release from triglycerides, which could stimulate mitochondrial respiration and thermogenesis. It is possible that the moderate increase of mitochondrial function could be a collective effect from both mechanisms and act downstream of the increased lipolysis to enhance energy expenditure.

Interestingly, our study found that whole‐body knockout of caspase‐6 does not enhance adipogenesis like TZDs, despite stabilizing and increasing PPARγ protein. To understand why increased PPARγ protein does not recapitulate the strong adipogenic response seen with TZDs, it is important to consider how PPARγ activation is regulated. TZDs are synthetic PPARγ agonists that are substantially more potent than most endogenous ligands. Rosiglitazone has an EC50 of ∼60 nM and robustly induces differentiation of pluripotent C3H10T1/2 stem cell into adipocytes in vitro. In the absence of pharmacological activation, PPARγ activity in preadipocytes is determined by PPARγ protein abundance and the availability/activity of endogenous ligands. Also, the function of PPARγ is regulated by covalent modification and its interactions with other co‐factors. A previous study by Qiang et al. uncovered that SirT1‐mediated deacetylation activates PPARγ and induces its interaction with PRDM16, thus promoting browning/beiging of white adipocyte without affecting adipogenesis [29], highlighting the complexity of PPARγ regulation. In our study, although caspase‐6 deletion mitigates HFD‐induced reductions in PPARγ protein and enhances ATGL‐mediated lipolysis and energy expenditure, it does not appear to promote adipogenesis. This is possibly due to the increase of SP1, which acts as a cofactor of PPARγ. Prior work showed that SP1 attenuates PPARγ‐induced ATGL expression in preadipocytes, but is required for PPARγ‐induced ATGL expression in mature adipocytes [23] (Figure 5J), suggesting a complex interplay between PPARγ and SP1. Overall, our findings suggest that caspase‐6 may represent an attractive therapeutic target for obesity and related metabolic disorders, as it enhances PPARγ‐induced ATGL expression and energy expenditure without promoting adipose tissue expansion.

Protease‐mediated proteolytic cleavage and subsequent degradation represent a widely adopted strategy to regulate the activity of nuclear receptors. Multiple nuclear receptors have been reported to be regulated by proteolytic cleavage. It was shown that caspase‐1 cleaves glucocorticoid receptor (GR) to induce its degradation, resulting in glucocorticoid resistance in leukemia cells [68]. Cathepsin L was reported to cleave retinoid X receptor alpha (RXRα) to impair thyroid hormone response in hepatocytes, as RXRα functions as a cofactor of thyroid hormone receptor [69]. Calpain cleaves androgen receptor (AR) to mediate calcium‐induced AR breakdown in LNCaP prostate cancer cells [70]. Estrogen receptor (ER) and progesterone receptor (PR) were also reported to be cleaved by calpain [71, 72]. Extending this concept beyond prior cell‐based studies, our study using new transgenic mice and diet‐induced obesity models demonstrates the physiological relevance and functional impact of caspase‐6‐mediated PPARγ cleavage, establishing proteolytic regulation as a previously underappreciated yet fundamental mechanism governing nuclear receptor activity in vivo.

In summary, our studies reveal a novel Casp6‐PPARγ/SP1‐ATGL axis regulating lipid and energy homeostasis, shedding new light on the molecular mechanisms underlying metabolic dysfunction during diet‐induced obesity. We uncovered PPARγ and SP1 as direct substrates of caspase‐6, linking a classical cell death effector to metabolic regulation. Notably, transcriptomic data from human adipose tissues validated the inverse correlations between caspase‐6, and the PPARγ‐ATGL pathways, reinforcing the translational relevance of our findings. Given our prior demonstration that caspase‐6 inhibition improves liver injury and fibrosis in MASH [3], our study suggests that targeting caspase‐6 could provide a unified therapeutic strategy to treat obesity, insulin resistance, and MASH simultaneously.

Future Perspective: Our study utilized transcriptomic analyses across multiple adipose depots to identify pathways/targets regulated by caspase‐6 deficiency. However, to fully understand the functions of caspase‐6 at the molecular level, a comprehensive and unbiased proteomic approach will be needed to define the full spectrum of caspase‐6 targets. Such proteomic profiling represents an important future direction that could further expand the molecular mechanisms uncovered in the present study.

## 4 Experimental Section

### 4.1 Animals

All animal experiments were conducted in accordance with institutional guidelines and were approved by the Institutional Animal Care and Use Committee of University of Texas Health science Center San Antonio (Approved protocol number: 20200115AR). Mice were housed under controlled conditions with an ambient temperature and a 12‐hour light/dark cycle with ad‐libitum access to food and water. All mouse models used in the study were on C57BL/6J background, including newly generated Caspase‐6 Flox mouse, which were backcrossed with C57BL/6J mice for at least 8 generations. Male mice were used for mouse models, while female mice were used for the isolation of primary cells or tissues for in vitro and *ex vivo* studies. For mice of different genotypes/groups, littermates and cagemates were used for experimental cohorts. Pups were randomly assigned to separate cages at weaning without genotype information. Entire cages were used for experiments. Diet feeding was initiated in mice between 10 and 14 weeks of age, with most studies starting at approximately 12 weeks. All phenotypic studies were performed in a blinded manner. Chow Diet vs. HFD‐fed mice: 12‐week‐old C57BL/6J mice were fed with either chow diet or 60% high fat diet (HFD) (Research diet #D12492). After 12 weeks of feeding, the mice were fasted 10 hrs before being euthanized with carbon dioxide. Epididymal adipose tissue, inguinal adipose tissue, brown adipose tissue, and liver were weighted, and snap‐frozen for analysis. Caspase‐6 KO mice: Caspase‐6 KO mice were obtained from The Jackson Laboratory (Stock# 006236). WT and Caspase‐6 KO mice were generated by breeding heterozygous mice to heterozygous mice. Flox and adipocyte‐specific caspase‐6 KO mice: Caspase‐6 Flox mice were generated by Ingenious Targeting Laboratory. Exon 5 of *Casp6* gene was flanked by two LoxP sites in Flox mice. To generate adipocyte‐specific caspase‐6 knockout mice, Flox mice were bred with C57BL/6‐Tg(Adipoq‐iCre/ERT2)1Soff/J mice (Strain# 025124, The Jackson laboratory). 12‐week‐old both Flox and ACKO were injected with 1 mg tamoxifen dissolved in corn oil (i.p.) for 5 consecutive days. One week after injection, both Flox and ACKO mice were fed a chow diet or 60% high‐fat diet for 12 weeks. At the conclusion of the study, mice were fasted 10 hrs, euthanized with carbon dioxide and then sacrificed for blood and tissue collection. Body composition analysis: Body composition analysis was performed with EchoMRI 3‐in‐1 system (Echo Medical Systems Ltd.).

### 4.2 Insulin Tolerance Test

Mice were fed chow diet or 60% HFD for 12 weeks, and fasted for 3 h before performing insulin tolerance test (ITT). After basal blood glucose levels were measured from tail‐vein blood, mice were intraperitoneally injected Humulin R at a dose of 0.75 IU/kg (chow diet‐fed mice) or 1 IU/kg (HFD‐fed mice). Blood glucose levels were subsequently measured at 15, 30, 45, 60, 90, and 120 min post‐injection using an EasyTouch blood glucose meter.

### 4.3 Glucose Tolerance Test

Mice were fed chow diet or 60% HFD for 12 weeks, and fasted for 10 hrs before performing glucose tolerance test (GTT). Fasting blood glucose levels were measured from tail‐vein blood. Mice were then intraperitoneally injected glucose at a dose of 2 g/kg (chow diet‐fed mice) or 1.2 g/kg (HFD‐fed mice). Blood glucose levels were then measured at 15, 30, 45, 60, 90, and 120 min post‐injection using an EasyTouch blood glucose meter.

### 4.4 Indirect Calorimetry

Promethion Core Behavioral System for Mouse (Sable Systems International) was used for indirect calorimetry study. Each mouse was singly housed under ambient temperature with free access to food and water. Mice were acclimatized to the environment for two days before data collection. Data were recorded to analyze oxygen consumption rate (VO_2_), carbon dioxide production rate (VCO_2_), energy expenditure, respiratory exchange rate (RER), physical activity, and food consumption. ANCOVA (body weight as a covariate) was used to assess the difference between the groups in VO_2_, VCO_2_, and energy expenditure.

### 4.5 Caspase‐6 Activity Measurement

Abcam Caspase‐6 Assay Kit (Cat No. ab39709) was used to measure the activity of caspase‐6 in adipose tissue following manufacturer's instructions.

### 4.6 Cell Culture

3T3‐L1 preadipocytes and isolated mouse primary preadipocytes were used in this study. 3T3‐L1 preadipocytes (originally from Howard Green Lab) were cultured in DMEM supplemented with 10% newborn calf serum. Cells were seeded in cell culture plates and grown until full confluence. Upon confluence, the medium was replaced with DMEM supplemented with 10% fetal bovine serum. For adipocyte differentiation, cells were induced with 0.5 mM IBMX, 250 nM dexamethasone, and 1 µg/ml insulin for 4 days. The medium was then replaced with 10% fetal bovine serum DMEM with 1 µg/ml insulin for 2–3 days to achieve full differentiation into mature adipocytes. Mouse primary preadipocytes were isolated from inguinal adipose tissue or brown adipose tissue. Tissues were collected, minced in a sterile petri dish, and digested with 2 mg/ml collagenase at 37°C. The digestion was stopped by adding fetal bovine serum. The cell suspension was then passed through a 100 µm cell strainer, and the flow‐through was washed twice with sterile phosphate‐buffered saline (PBS). Mouse primary preadipocytes were differentiated into mature adipocytes following the same protocol as 3T3‐L1 preadipocyte differentiation with the addition of 1 µM rosiglitazone.

### 4.7 Protein Isolation and Western Blotting

Cells were briefly washed with cold PBS and collected in cell lysis buffer (G‐Bioscience #786‐180) supplemented with a protease inhibitor cocktail (Roche #11836170001). The protein samples were then resolved on Bio‐Rad precast gels and transferred to an activated PVDF membrane. The membrane was blocked with 5% skimmed milk powder for 1 h before incubation with the primary antibody in a 2% BSA solution. This was followed by incubation with the secondary antibody. The membranes were developed using a chemiluminescence reagent (Millipore Sigma, WBKLS0500). The list of antibodies used is as follows, all the primary antibodies were diluted 1:1000 for use in western blotting.

Caspase‐6 Cell signaling technology#9762
Active caspase‐6 Cell signaling technology#9761
ATGL Cell signaling technology#2138
Beta actin Life Technologies#MA5‐32540
PPARγ Santa Cruz Biotechnology#Sc‐7273
SP1 Santa Cruz Biotechnology#Sc‐17824
OXPHOS Rodent WB cocktailThermo Fisher Scientific #45‐8099

### 4.8 RNA Isolation and Realtime qPCR

RNA was isolated from either cultured cells or animal tissues. For cultured cells, lysis was performed using the lysis buffer from the PureLink RNA Mini Kit (Life Technologies #12183025), and RNA was isolated following the manufacturer's instructions. For RNA isolation from mouse tissues, the tissues were homogenized in TRIzol reagent (Thermo Fisher Scientific #15596026) using a bead‐beater. Chloroform (20%) was added to the mixture, followed by vortex and centrifugation. The upper layer containing RNA was collected and further purified using the PureLink RNA Mini Kit according to the manufacturer's instructions. 1 µg of RNA was used to synthesize cDNA using 5X PrimeScript RT Master Mix (Takara #RR036A‐1) following the manufacturer's instructions. The resulting cDNA was then used for real‐time qPCR with PowerTrack SYBR Green Master Mix (Applied Biosystems #C14512). qPCR data were recorded using the CFX Opus 384 Real‐Time PCR System (Bio‐Rad Laboratories) and analyzed with Bio‐Rad CFX Maestro software. Primer sequences used for gene expression analysis are as follows:

**Table:** 

Gene Name	Forward Primer	Reverse Primer
*Casp6*	CATGGTGGATCACCAGACAGAC	GGAGCCATTCACAGTTTCTCGG
*Rplp0*	GCAGACAACGTGGGCTCCAAGCAGAT	GGTCCTCCTTGGTGAACACGAAGCCC
*Il6*	TAGTCCTTCCTACCCCAATTTCC	TTGGTCCTTAGCCACTCCTTC
*Il1b*	GCAACTGTTCCTGAACTCAACT	ATCTTTTGGGGTCCGTCAACT
*Tnf*	CCCTCACACTCAGATCATCTTCT	GCTACGACGTGGGCTACAG
*Ifng*	ATGAACGCTACACACTGCATC	CCATCCTTTTGCCAGTTCCTC
*Il10*	GCTCTTACTGACTGGCATGAG	CGCAGCTCTAGGAGCATGTG
*Atp5f1b*	GGTTCATCCTGCCAGAGACTA	AATCCCTCATCGAACTGGACG
*Cox4i1*	ATTGGCAAGAGAGCCATTTCTAC	CACGCCGATCAGCGTAAGT
*Ndufv1*	TTTCTCGGCGGGTTGGTTC	GGTTGGTAAAGATCCGGTCTTC
*Ppargc1a*	TATGGAGTGACATAGAGTGTGCT	CCACTTCAATCCACCCAGAAAG
*Ucp1*	AGGCTTCCAGTACCATTAGGT	CTGAGTGAGGCAAAGCTGATTT
*Uqcrc2*	AAAGTTGCCCCGAAGGTTAAA	GAGCATAGTTTTCCAGAGAAGCA
*Pnpla2*	GGATGGCGGCATTTCAGACA	CAAAGGGTTGGGTTGGTTCAG

Table 1. QPCR primer sequences

### 4.9 Lipid Measurements

#### 4.9.1 Triglycerides

Triglyceride levels in mouse serum samples were estimated using the Triglyceride Quantification Kit (Abcam #ab65336) following the manufacturer's instructions. Free Glycerol: Free glycerol levels in mouse serum or cell culture supernatant were measured using the Free Glycerol Reagent (Sigma‐Aldrich #F6428) according to the manufacturer's manual. Non‐Esterified Fatty Acids (NEFA): NEFA levels in cell culture supernatant were determined using the HR Series NEFA‐HR Kit (Fujifilm #999‐34691, #991‐34891, #995‐34791, #993‐35191), following the manufacturer's manual.

### 4.10 Insulin ELISA

Mouse serum insulin levels were measured using the Ultra‐Sensitive Mouse Insulin ELISA Kit (Crystal Chem #90080) following the manufacturer's instructions. Briefly, 5 µL of mouse serum sample or insulin standard was mixed with 95 µL of sample diluent and added to the precoated plate. After 2 h of incubation at 4°C, the plate was washed, and 100 µL of conjugate solution was added to each well, followed by a 30‐min incubation at room temperature. The plate was then washed again, and 100 µL of substrate solution was added to each well, incubated for 20 min at room temperature, and then 100 µL of stop solution was added. The absorbance was recorded at 450/630 nm, and absolute insulin levels were calculated accordingly.

### 4.11 Seahorse Assay

Cell oxygen consumption rate (OCR) was measured using the Agilent Seahorse XFe96 Analyzer [73]. Mouse primary pre‐adipocytes were isolated form the inguinal adipose tissue of wild type or caspase‐6 KO mice. Cells were seeded in the XFe96 cell culture plate and differentiated into mature adipocytes following the differentiation protocol mentioned earlier. Post differentiation, cells were used to assess the oxygen consumption rate. One day before the experiment, the sensor cartridge was calibrated overnight with Seahorse Assay Buffer in a non‐CO_2_ incubator at 37°C. On the day of the experiment, the cell culture plate was washed twice with Seahorse Assay Media supplemented with 25 mM glucose and 1 mM pyruvate, then incubated in the same supplemented media for 1 h at 37°C in a non‐CO_2_ incubator. Simultaneously, the cartridge was loaded with oligomycin (2 µM) (Sigma Aldrich #O4876‐5 g), FCCP (5 µM) (SML‐2959‐1 mL), rotenone (8 µM) (Sigma Aldrich #R8875‐1 g) and antimycin A (8 µM) (Sigma Aldrich #A8674‐25 mg). The Mito Stress Test assay was initiated to calibrate the cartridge. The cell plate was then placed into the analyzer, and OCR measurements were recorded. Following the experiment, protein content was measured and utilized for normalization.

### 4.12 In Vitro Cleavage Assay

Recombinant active caspase‐6 was obtained from Enzo Life Sciences (#BML‐SE170), recombinant PPARγ from Sino Biological (#12019‐H20B), and recombinant SP1 from Active Motif (#81181). The 2x cleavage buffer consist of 100 mM HEPES, 6 mM EDTA, 300 mM NaCl, and 0.01% Tween‐20. For in vitro cleavage assay, 3 µg of substrate protein (PPARγ or SP1) was mixed with 1U of active caspase‐6, 10 mM DTT, and 50% 2x cleavage buffer. The total reaction volume was adjusted to 25 µl. The mixture was incubated at 37°C for 1 h.

### 4.13 Lipidomic Analysis for Triglyceride and FAHFA Species

Triglyceride and FAHFA species were analyzed using multidimensional mass spectrometry‐based shotgun lipidomic analysis [74]. The protein concentration of individual adipose sample was quantified using the Pierce BCA protein assay kit (Cat #23225, Thermo Scientific). Bovine serum albumin was used as standard. An adequate amount of each adipose tissue homogenate (equivalent to 0.2 mg protein) was accurately transferred into a disposable glass culture test tube. A premixture of lipid internal standard (IS) species, including T17:1 triacylglycereol (TG), 12‐d4‐16:0H18:0 fatty acid esters of hydroxy fatty acid (FAHFA), was added prior to conducting lipid extraction for quantification of the targeted classes of lipid species [75]. Lipid extraction was performed using a modified Bligh and Dyer procedure [74]. The organic phase was collected into a new glass tube, and then dried with N_2_ steam to minimize oxidation and preserve lipid integrity. after extraction, lipid extracts were resuspended in a chloroform (CHCl_3_): methanol (MeOH) mixture (1:1, v/v) at 400 µL/mg of protein. Aliquot 10 µL and 50 µL of individual lipid extract were used to perform derivatization for FAHFA [76] analysis, respectively. The TG analysis was analysis as described previously [77]. The lipid extracts were stored at ‐80°C till analysis.

*Derivatization of FAHFA species* Briefly, resuspended the lipid extract with 200 µL of CHCl_3_ and vortexed for 10 sec. Conditioned a SPE column (200 mg) with 15 mL of hexane, allow solvent to flow through the column by gravity. After hexane totally flowing through the column, load the 200 µL of sample onto the column. Washed the neutral lipids with 16 mL of 5% ethyl acetate in hexane. Then the FAHFA was eluted with 16 mL of ethyl acetate and dried under N_2_ stream. After evaporation of the solvent in a lipid solution under a N_2_ stream, added 10 µL of ice‐cold acetonitrile/N,N‐dimethylformamide (4:1, *v/v*) and 640 mM [3‐(dimethylamino)propyl]‐ethylcarbodiimide hydrochloride aqueous solution, respectively, into the conical tube containing the lipid residue. Briefly vortexed to mix the solutions and lipid residue, then added 10 µL of 10 mM N‐hydroxybenzotriazole in acetonitrile and 10 µL of 30 mM N‐[4‐(aminomethyl)phenyl]pyridinium in acetonitrile, respectively. The tube containing mixed solution was thoroughly vortexed, filled with nitrogen, capped, and incubated at 70°C for 90 min. After incubation, the derivatives were extracted with 4.5 mL of CHCl_3_/MeOH/distilled water (1:1:1, *v/v/v*). The bottom layer was collected, and the solvents were evaporated under a N_2_ stream. The residue was resuspended in 100 µL of CHCl_3_/MeOH (1:1, *v/v*) for mass spectrometric analysis.

For shotgun lipidomics, lipid extract was further diluted to a final concentration of ∼400 fmol total lipids per µL. Data acquisition for TG and FAHFA was performed on a TSQ Altis triple quadrupole mass spectrometer (Table1. TSQ Altis, Thermo Fisher Scientific, San Jose, CA), which was equipped with an automated nanospray device (TriVersa NanoMate, Advion Bioscience Ltd., Ithaca, NY) as described [78]. Internal standards were applied to correct extraction efficiency and matrix effects. Identification and quantification of lipid species were performed using an in‐house automated software program [79, 80]. Data processing (e.g., ion peak selection, baseline correction, data transfer, peak intensity comparison, and quantitation) was performed as described [80]. Details on the linear dynamic range, signal‐to‐noise threshold, and quality control procedures followed previously established protocols [74, 78]. The result was normalized to the protein content (nmol lipids/mg protein).

Table 2. MS scan modes and method information for analysis of lipid species

**Table:** 

Lipid Class	Polarity	Collision Energy [V]	Scan Model
TG	Positive	35	Neutral Loss Scan (228.2, 254.2, 256.2, 268.2, 278.2, 280.2, 282.2, 284.3, 302.2, 304.2, 306.2, 308.3, 310.3, 312.3, 328.2, 330.2, 332.2, 334.3, 336.3, 338.3, 340.3)
FAHFA	Positive	52	Neutral Loss Scan (254.2, 256.2, 260.3, 280.2, 282.2, 284.3)

### 4.14 RNA Sequencing

#### 4.14.1 Library Preparation

Tissues were homogenized in TRIzol reagent and total RNA was isolated using PureLink RNA Mini Kit following manufacturer's instructions (Life Technologies). RNA quality was assessed by Agilent 4200 TapeStation System. RNA‐seq libraries were prepared from poly(A)‐enriched mRNA as previously described [81, 82, 83, 84]. Libraries were then size selected and purified by SpeedBeads, quantified using a Qubit dsDNA HS Assay Kit (Thermo Fisher Scientific), and sequenced on a NextSeq 2000 platform (Illumina, San Diego, CA) at the Genome Sequencing Facility of the University of Texas Health Science Center at San Antonio.

#### 4.14.2 RNA Sequencing Analysis

RNA‐seq analysis was performed following established protocols [81, 83, 85]. Sequencing data in FASTQ format were mapped to the mouse mm10 genome using STAR with default parameters [85]. To generate raw read counts, HOMER's analyzeRepeats.pl with the option rna and the parameters ‐condenseGenes and ‐count exons were used on three replicates per condition. Each sequencing experiment was normalized to a total of 10^7^ uniquely mapped tags by adjusting the number of tags at each position in the genome to the correct fractional amount given the total tags mapped. To generate TPM values, the parameters ‐count exons ‐condenseGenes ‐tpm were used. The TPM values were further processed by log_2_(TPM+1). Differential gene expression between specified groups was assessed with DESeq2 using HOMER's getDiffExpression.pl with the parameters p‐adj < 0.05 and FC (fold change) ≥ 1.5, utilizing three replicates per condition [82, 83, 84, 85, 86]. Functional enrichment and ontology analyses were carried out using KEGG and Metascape [83, 87].

### 4.15 Analysis of Published Datasets

Publicly available and anonymized RNA‐seq datasets were obtained from the GEO. The first dataset, GSE59034, contains human subcutaneous white adipose tissue transcriptomes from anonymized never obese donors (n = 16) and obese subjects prior to bariatric surgery (n = 16). The second dataset, GSE245948, includes human subcutaneous adipose depots from anonymized individuals with obesity (n = 76). The third dataset, GSM2420483, consists of inguinal white adipose tissue from C57BL/6J mice fed a 60% high fat diet for 12 weeks, with or without rosiglitazone treatment (3.6 mg/kg/d during the final 2 weeks). Normalized gene expression values and accompanying statistical metrics provided within each dataset were used to quantify transcript abundance and assess the relationship of *CASP6* expression with BMI and established PPARγ target genes.

### 4.16 PPARγ Reporter Assay

HEK293T cells were cultured and seeded into a 96‐well clear‐bottom imaging plate. Once the cells reached approximately 70% confluency, they were transfected with the PPRE‐H2B‐eGFP reporter construct (Addgene #84393), which express a fluorescent reporter of PPARγ activity, along with either WT PPARγ or PPARγ D69E mutant plasmids using Lipofectamine 3000.40 h after transfection, half of the wells from each condition were treated with CHX‐TNFα in phenol red–free DMEM. Fluorescence intensity was measured at baseline (0 h) and again after 8 h of treatment. The recorded values were normalized and presented as percentage of change in fluorescence intensity to indicate PPARγ activity.

### 4.17 Site‐Directed Mutagenesis

Site‐directed mutagenesis was performed as previously described [3]. Flag‐PPARγ2 D/E and Myc‐SP1 D/E plasmids were generated using Kapa Hifi DNA polymerase with 0.5 ng WT plasmid as template DNA in 50ul reaction. Template DNA was digested by DpnI (NEB). Stellar competent cell (Takara) was used for transformation and plasmids amplification. Plasmid was purified with Wizard Plus SV Minipreps DNA purification system (Promega). Mutation was confirmed by Sanger sequencing.

### 4.18 Ex Vivo Study

EWAT was isolated from chow diet‐fed female WT and C6KO mice. After collection, tissues were washed thoroughly with PBS and transferred into DMEM with 10% FBS. The tissue was then minced into small pieces using sterile scissors. Equal amounts of tissue were placed into each cell culture plate containing DMEM with 10% FBS. 2 hrs before treatment, the culture medium was replaced with DMEM supplemented with 2% BSA. Then tissues were treated with or without CHX (15 µg/ml) and TNFα (75 ng/ml). Tissue samples were harvested after 6 or 24 h of treatment. Samples were then lysed for protein isolation. Western blotting was performed to determine levels of indicated proteins.

### 4.19 Statistics

Data are presented as mean ± SEM (in vivo study) or mean± SD (in vitro study). Replicates are indicated in figure legends. Statistical significance between two groups was determined using Student's *t*‐test. For the insulin tolerance test (ITT) and glucose tolerance test (GTT), a two‐way ANOVA was performed, followed by Šídák's‐corrected *post hoc* test for multiple comparisons test. ANCOVA analysis was used for indirect calorimetry using body weight as a covariate. Statistical significance was set at p < 0.05. GraphPad Prism 10 was used for statistical analysis.

## Author Contributions

P.Z. and X.S. conceived the project. P.Z., X.S., and A.G. designed the study and performed the experiments. X.S., W.Y., and J.J. performed RNA‐seq experiments and bioinformatic analysis. L.H. assisted with histology staining and imaging. M.P. and X.H. performed lipidomics and data analysis. A.G., P.Z., and X.S. wrote the manuscript. The final version was reviewed by all authors.

## Conflicts of Interest

P.Z. is a named inventor of a patent application related to the use of caspase‐6 as a therapeutic target for MASH.

## Supporting information




**Supporting File 1**: advs73758‐sup‐0001‐SuppMat.docx.

**Supporting File 2**: advs73758‐sup‐0002‐Table S1.xlsx.

**Supporting File 3**: advs73758‐sup‐0002‐Table S2.xlsx.

**Supporting File 4**: advs73758‐sup‐0003‐Table S3.xlsx.

**Supporting File 5**: advs73758‐sup‐0004‐Table S4.xlsx.

**Supporting File 6**: advs73758‐sup‐0006‐DataFile.pdf.

## Data Availability

The data that support the findings of this study are available from the corresponding author upon reasonable request. The RNAseq dataset can be accessed in GEO DataSets (GSE311559).
