# Supplementary material for: Caspase‐6 Controls Lipid and Energy Metabolism in Diet‐Induced Obesity
Source: Adv Sci (Weinh). 2026 Apr 13;13(21):e14784. doi: 10.1002/advs.202514784 (PMC13073318; doi:10.1002/advs.202514784)
Supplement: Supplementary file 1 — Supporting File 1: advs73758‐sup‐0001‐SuppMat.docx. [file ADVS-13-e14784-s006.docx]

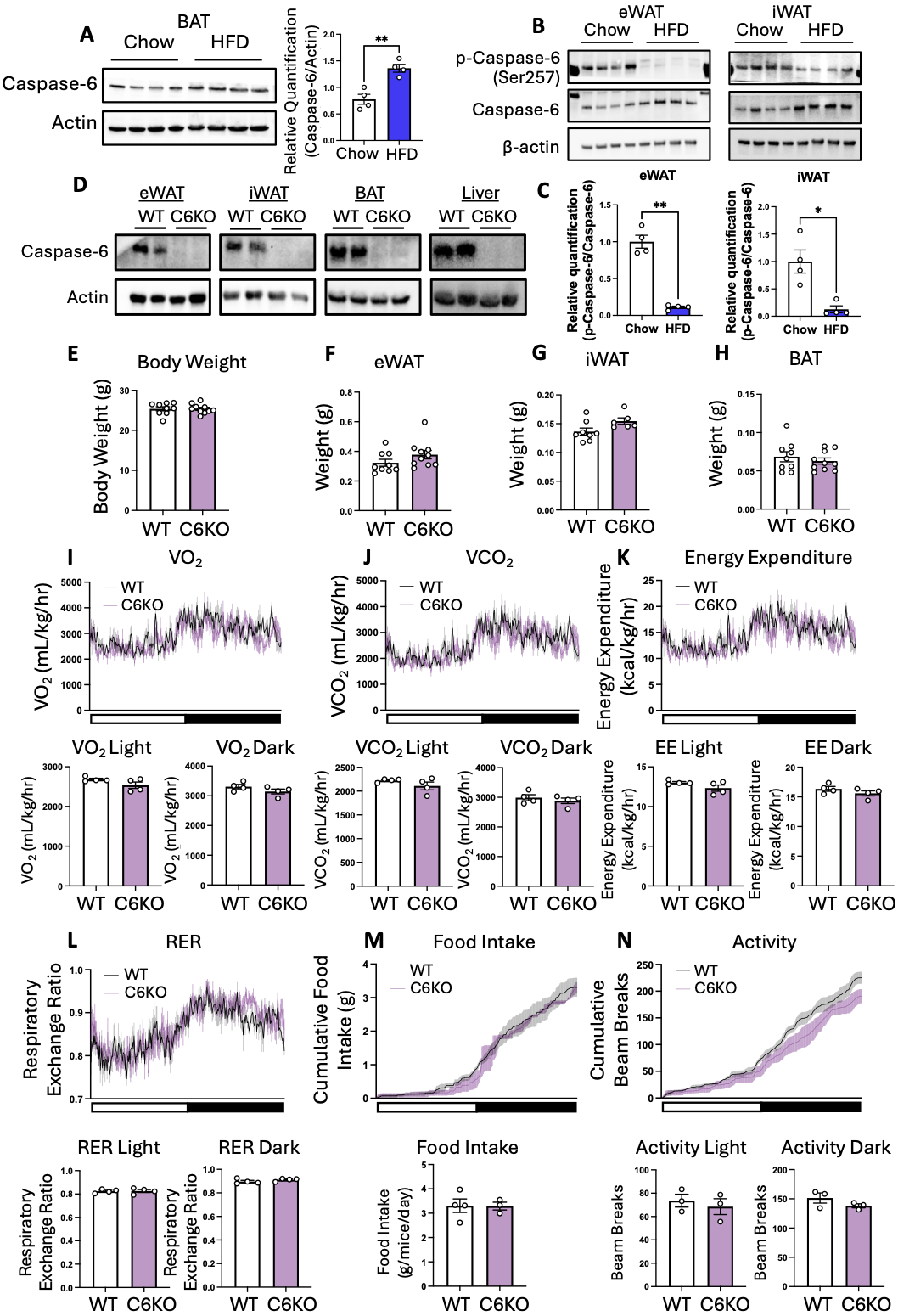


**Supplemental Figure 1. Caspase-6 deficiency does not alter energy metabolism in mice fed chow diet. A**. Immunoblot of caspase-6 protein and quantification in BAT of mice fed chow diet or HFD for 12 weeks (n=4). Two tailed unpaired Student’s *t*-test. **B-C.** Immunoblot (**B**) and quantification (**C**) of phospho-Ser^257^ Caspase-6 in eWAT and iWAT of mice fed chow diet or HFD for 12 weeks. Two tailed unpaired Student’s *t*-test. **D.** Immunoblot of caspase-6 protein in eWAT, iWAT, BAT, and liver of WT and C6KO mice. **E-N**. WT and C6KO mice fed chow diet for 12 weeks. **E.** Body weight (n=9-10). (**F-H**) Tissue weight (n=9-10): eWAT **(F**), iWAT **(G**), and BAT **(H**). Two tailed unpaired Student’s *t*-test. (**I-N**) Indirect calorimetry (n=4): (**I**) oxygen consumption rate, (**J**) carbon dioxide production, (**K**) energy expenditure, (**L**) respiratory exchange ratio (RER), (**M**) food intake. (**N**) Physical activity. (I-K) ANCOVA analysis with body weight as a covariate. (L-N) Two-way ANOVA followed by Šídák’s-corrected *post hoc* test. *p<0.05, **p<0.01, ***p<0.001, ****p<0.0001.

**
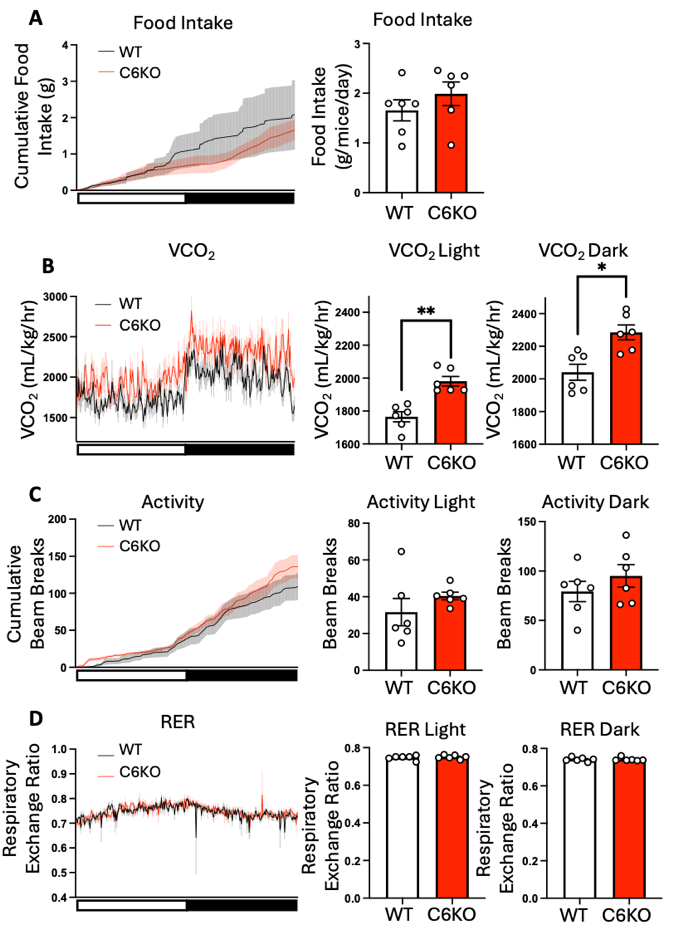
Supplemental Figure 2. Caspase-6 knockout alters energy metabolism in diet-induced obesity.** WT and C6KO mice fed HFD for 12 weeks. Indirect calorimetry (n=7): **A**. Food intake, **B**. carbon dioxide production, **C**. activity, **D**. RER. Data show Mean ± SEM; (A, C, D) Two-way ANOVA followed by Šídák’s-corrected *post hoc* test. (B) ANCOVA analysis with body weight as a covariate. *, p<0.05.


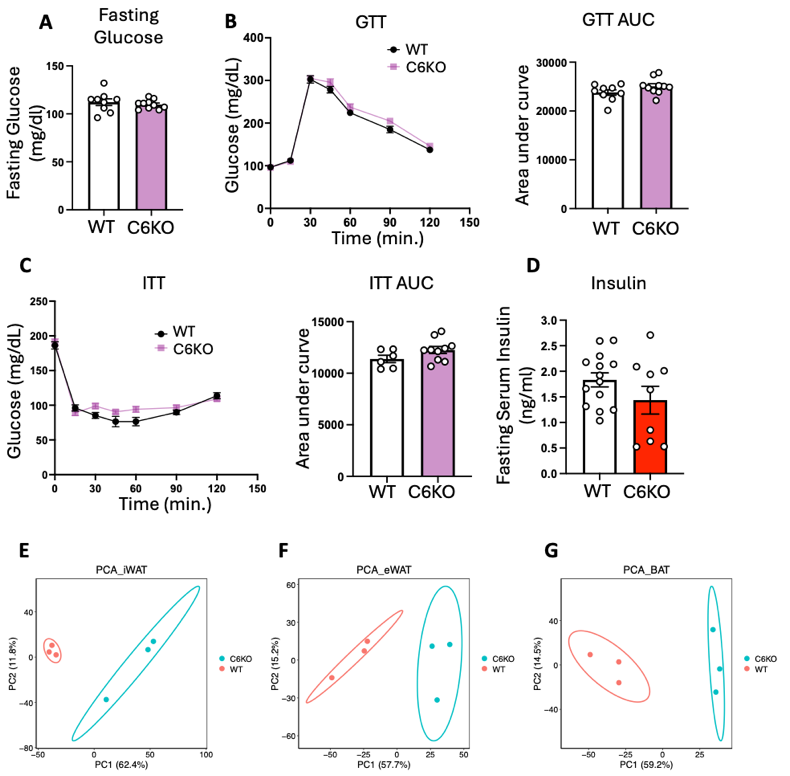


**Supplemental Figure 3. Caspase-6 deficiency does not affect glucose metabolism in chow diet-fed mice. A-C**. WT and C6KO mice fed chow diet for 12 weeks. (**A**) Fasting blood glucose (n=9-10). Two tailed unpaired Student’s *t*-test. (**B**) GTT and AUC quantification (n=9-10). (**C**) ITT and AUC quantification (n=6-10). GTT and ITT: two-way ANOVA followed by Šídák’s-corrected *post hoc* test. **D**. Fasting insulin levels in HFD-fed mice (n=9-13). Two tailed unpaired Student’s *t*-test. **E-G.** PCA plots of transcripts with a mean expression value of at least 4 normalized tags in at least one group, and differing in expression by at least 1.5-fold in comparison between WT and C6KO mice fed HFD for 12 weeks. Shown are PCA plots for iWAT (**E**), eWAT (**F**), and BAT (**G**). *p<0.05, **p<0.01, ***p<0.001, ****p<0.0001.


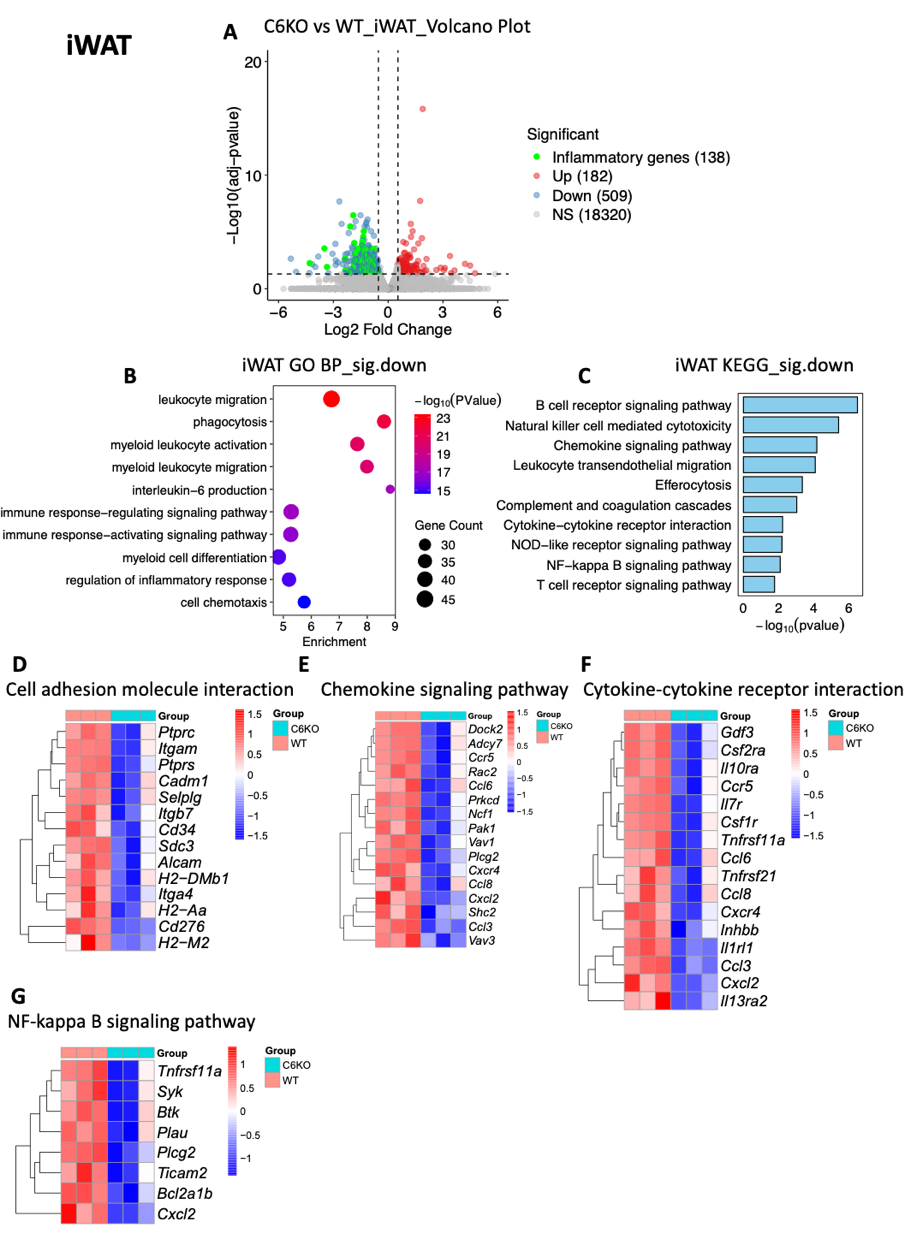


**Supplemental Figure 4. Transcriptomic analysis of iWAT from HFD-fed WT and C6KO mice.** Transcriptomic analysis of iWAT from WT and C6KO mice fed HFD for 12 weeks. **A**. Volcano plot showing differentially expressed genes (DEGs), with inflammatory genes highlighted in light green. **B**. Top 10 GO biological process terms for significantly downregulated genes. **C**. Top 10 KEGG pathways for downregulated genes. **D-G**. Relative expression values (Z-scaled log2 [TPM+1]) of downregulated genes in cell adhesion molecules interaction, chemokine signaling pathway, cytokine-cytokine receptor interaction and NF-κB signaling pathways.


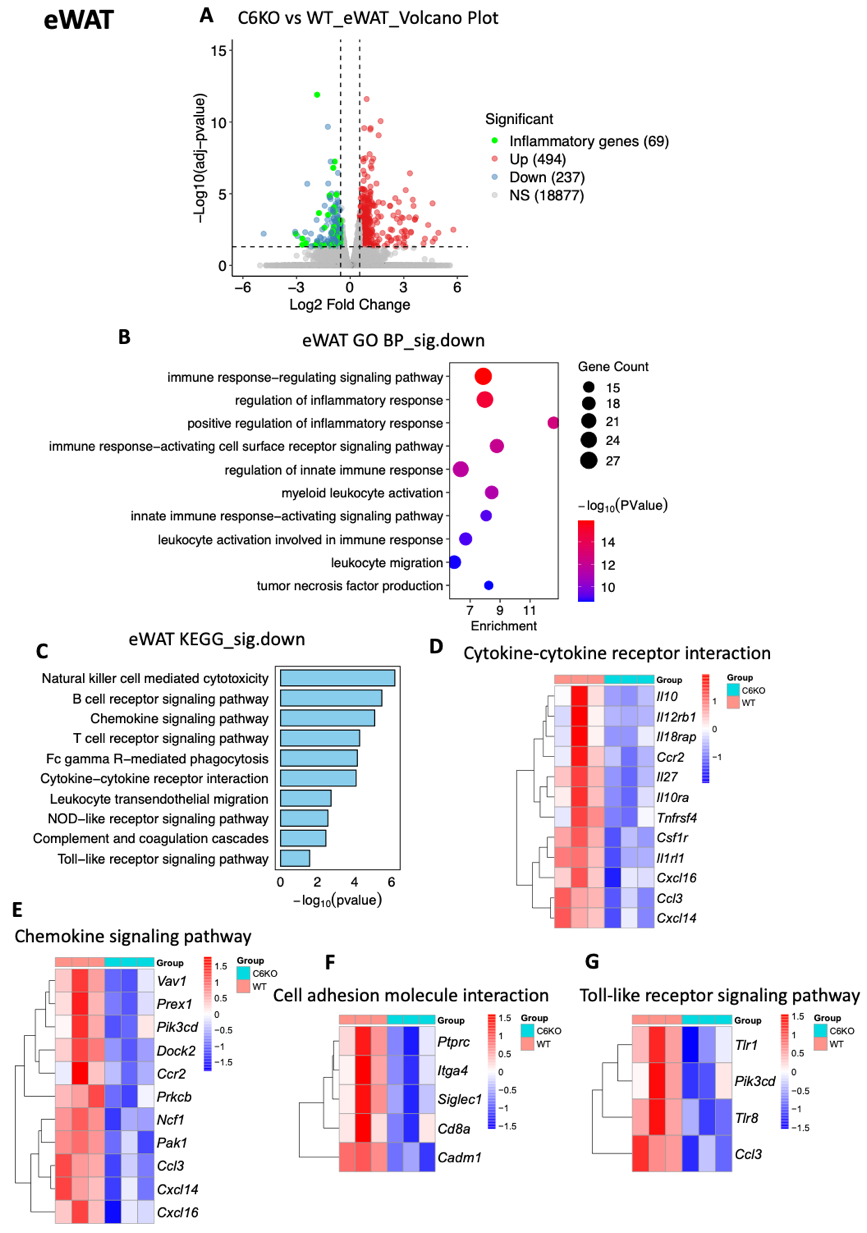


**Supplemental Figure 5. Transcriptomic analysis of eWAT from HFD-fed WT and C6KO mice.** Transcriptomic analysis of eWAT from WT and C6KO mice fed HFD for 12 weeks. **A**. Volcano plot showing DEGs, with inflammatory genes highlighted in light green. **B**. Top 10 GO biological process terms in downregulated genes. **C**. Top 10 KEGG pathways for downregulated genes. **D-G**. Relative expression values (Z-scaled log2 [TPM+1]) of downregulated genes in cytokine-cytokine receptor interaction, chemokine signaling pathway, cell adhesion molecules interaction, and Toll-like receptor signaling pathways.


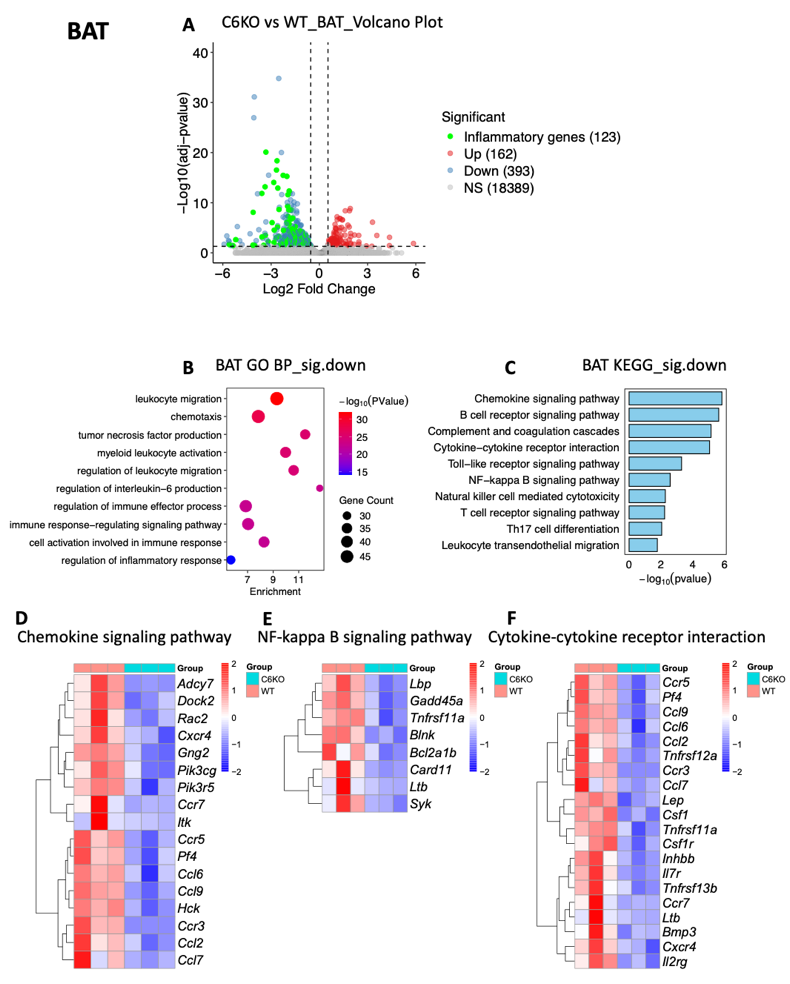


**Supplemental Figure 6. Transcriptomic analysis of BAT from HFD-fed WT and C6KO mice.** Transcriptomic analysis of BAT from WT and C6KO mice fed HFD for 12 weeks. **A**. Volcano plot showing DEGs, with inflammatory genes highlighted in light green. **B**. Top 10 GO biological process terms in downregulated genes. **C**. Top 10 KEGG pathways in downregulated genes. **D-F**. Relative expression values (Z-scaled log2 [TPM+1]) of downregulated genes in chemokine signaling pathway, NF-κB signaling pathway, and cytokine–cytokine receptor interaction pathways.


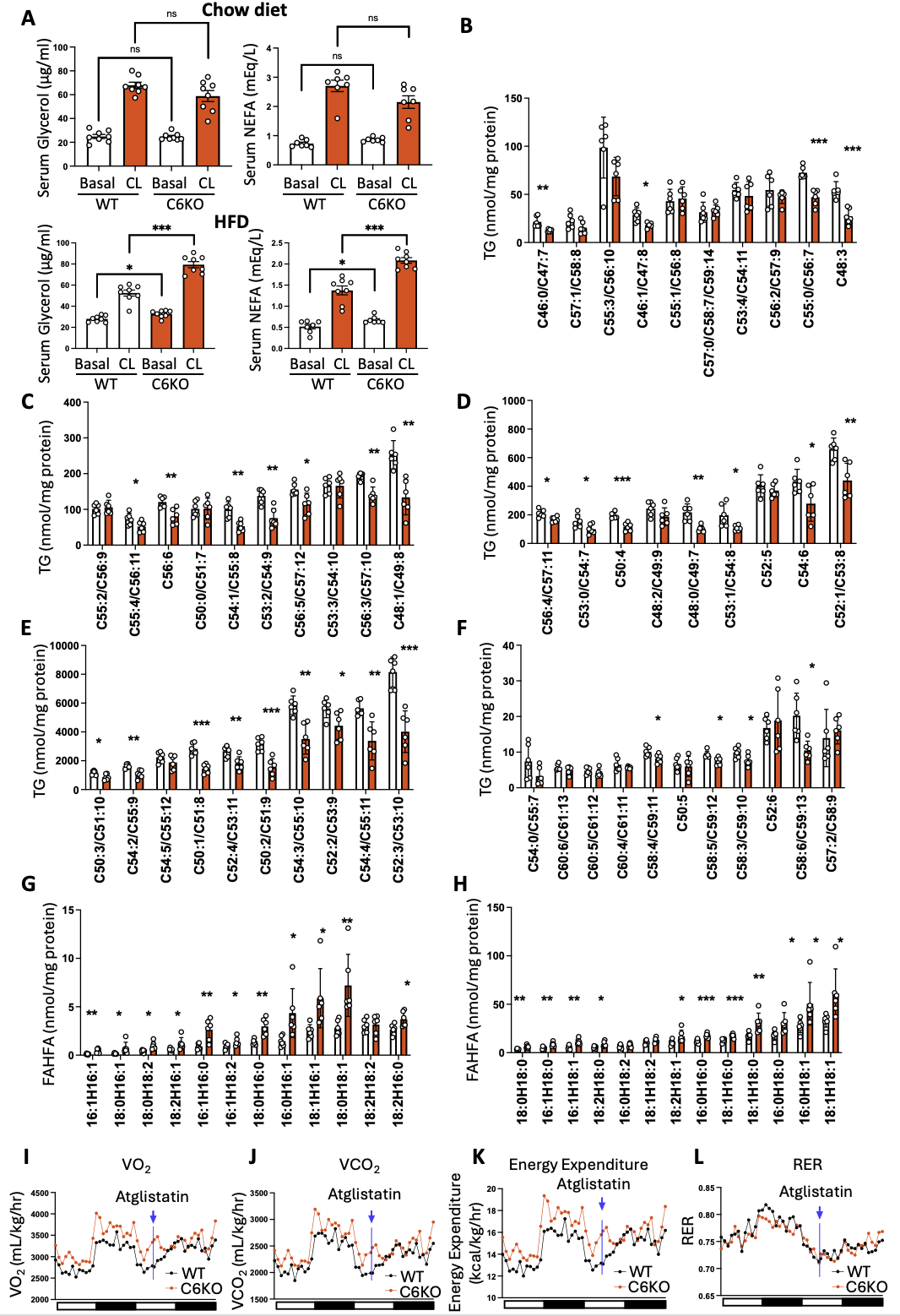


**Supplemental Figure 7. Lipid metabolism in adipose tissue from HFD-fed WT and C6KO mice. A.** In vivo lipolysis analysis in WT and C6KO mice fed chow diet or HFD for 10 weeks, fasted for 5hrs, and i.p. injected PBS or 0.5mg/kg CL-316,243 for 30min. Free Glycerol and NEFA were measured in mouse serum**.** Two-way ANOVA followed by Šídák’s-corrected *post hoc* test. **B-H** Lipidomic analysis of eWAT from WT and C6KO mice fed HFD for 12 weeks (n=6). **B-F**. Triglyceride species. **G-H**. FAHFA species. For each species: two tailed unpaired Student’s *t*-test. **I-L.** WT and C6KO mice fed HFD for 12 weeks. Indirect calorimetry on mice injected Atglistatin (n=3): oxygen consumption rate (**I**), carbon dioxide production (**J**), energy expenditure (**K**), and RER (**L**). ANCOVA analysis with body weight as a covariate. *p<0.05, **p<0.01, ***p<0.001, ****p<0.0001.


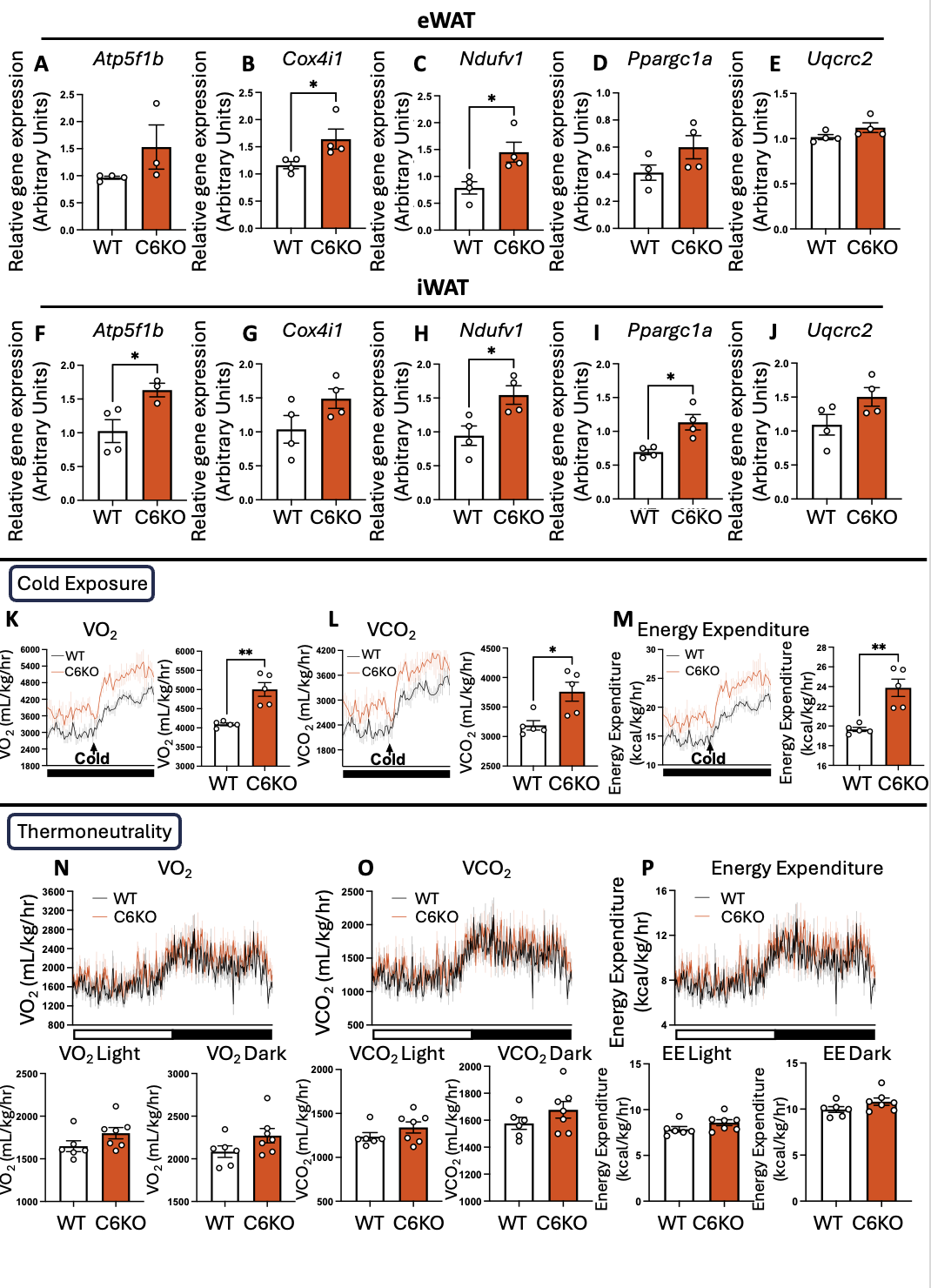


**Supplemental Figure 8. Caspase-6 deficiency modulates mitochondrial function. A-J.** WT and C6KO mice fed HFD for 12 weeks. A**-E**. Expression of mitochondrial markers in eWAT (n=4). **F-J**. Expression of mitochondrial markers in iWAT (n=4). Two tailed unpaired Student’s *t*-test. **K-M** WT and C6KO mice fed HFD for 12 weeks followed by 4°C cold exposure. Indirect calorimetry (n=5): oxygen consumption rate (**K**), carbon dioxide production (**L**), energy expenditure (**M**). ANCOVA analysis with body weight as a covariate. **N-P.** WT and C6KO mice fed HFD for 10 weeks, then housed under thermoneutral conditions (30^o^C). Indirect calorimetry (n=6-7): oxygen consumption rate (**N**), carbon dioxide production (**O**), energy expenditure (**P**). ANCOVA analysis with body weight as a covariate. *p<0.05, **p<0.01, ***p<0.001, ****p<0.0001.


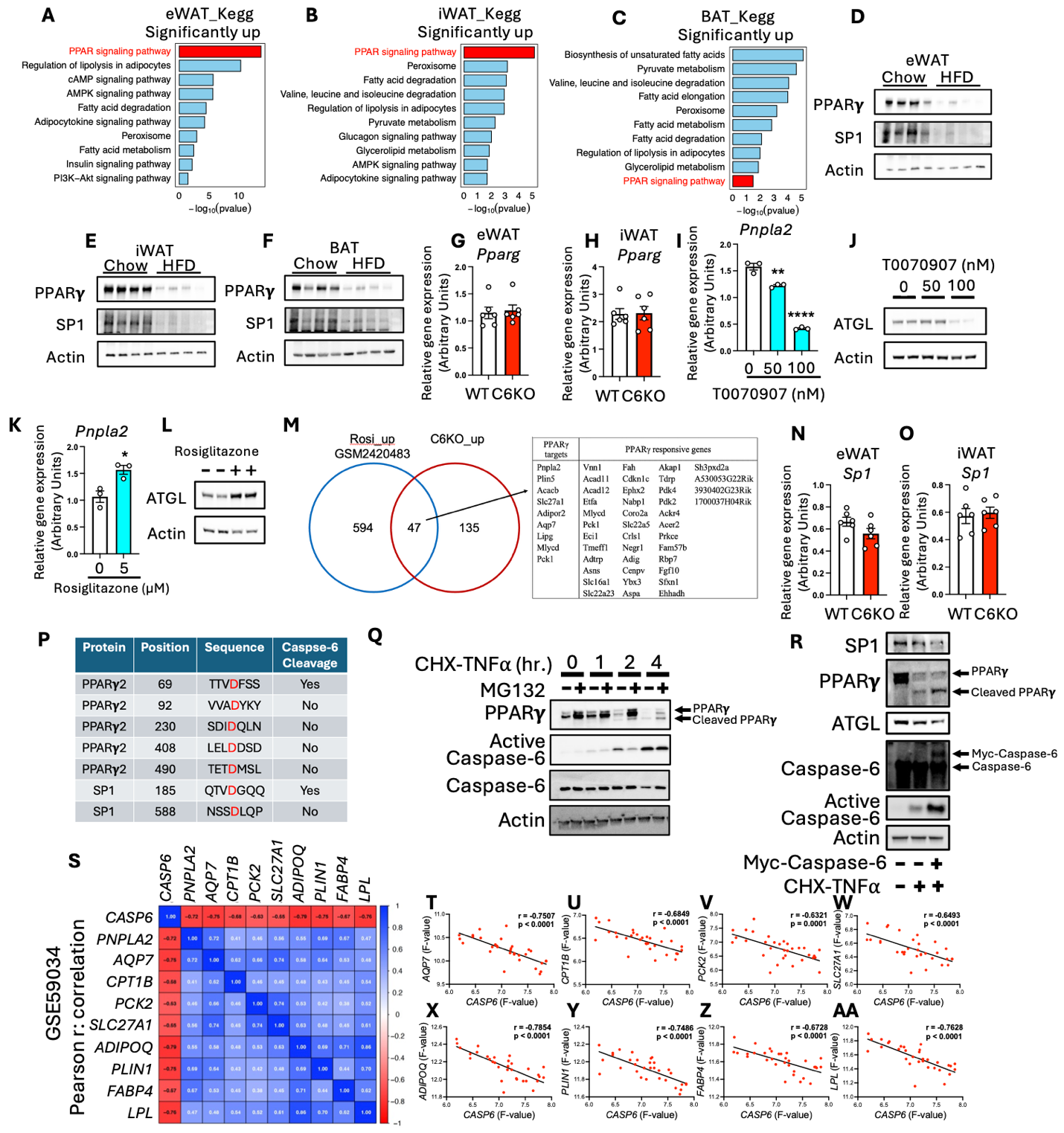


**Supplemental Figure 9. Caspase-6 cleaves PPARγ and SP1 to control ATGL expression. A-C**. KEGG pathway analysis of upregulated genes in RNAseq from eWAT (**A**), iWAT (**B**), and BAT (**C**) of C6KO mice fed HFD for 12 weeks. **D-F**. Immunoblot of PPARγ and SP1 proteins in eWAT (**D**), iWAT (**E**), and BAT (**F**) of mice fed chow diet or HFD for 12 weeks. **G-H.** Expression of *Pparg* in eWAT (**G**) and iWAT (**H**). Two tailed unpaired Student’s *t*-test. **I-J**. qPCR (**I**) and Immunoblot (**J**) of *Pnpla2* (ATGL) in 3T3-L1 adipocytes treated with T0070907 for 24hrs. Two tailed unpaired Student’s *t*-test. **K-L**. qPCR (**K**) and Immunoblot (**L**) of *Pnpla2* (ATGL) in 3T3-L1 adipocytes treated with rosiglitazone (5μM) for 24hrs. Two tailed unpaired Student’s *t*-test. **M.** Venn diagram showing genes upregulated by both C6KO and rosiglitazone treatment in adipose tissue of HFD-fed mice (RNAseq data of rosiglitazone-treated HFD-fed mice from GSM2420483). **N-O.** Expression of *Sp1* in eWAT (**N**) and iWAT (**O**). Two tailed unpaired Student’s *t*-test. **P**. Potential and identified caspase-6 cleavage sites in PPARγ2 and SP1. **Q**. Immunoblot of PPARγ and SP1 proteins in 3T3-L1 adipocytes treated with CHX (5μg/ml)-TNFα (25ng/ml) in the absence or presence of MG132 (20µM). **R.** Immunoblot of PPARγ, SP1 and ATGL in 3T3-L1 adipocytes overexpressing myc-caspase-6 treated with CHX (5μg/ml)-TNFα (25ng/ml). **S-AA**. Pearson correlation of *CASP6* and PPARγ target genes in human adipose tissue (GSE59034): **(S) Correlation matrix, (T)** *AQP7,* **(U)** *CPT1B,* **(V)** *PCK2,* **(W)** *SLC27A1,* **(X)** *ADIPOQ,* **(Y)** *PLIN1,* **(Z)** *FABP4, and* **(AA)** *LPL*. *p<0.05, **p<0.01, ***p<0.001, ****p<0.0001.


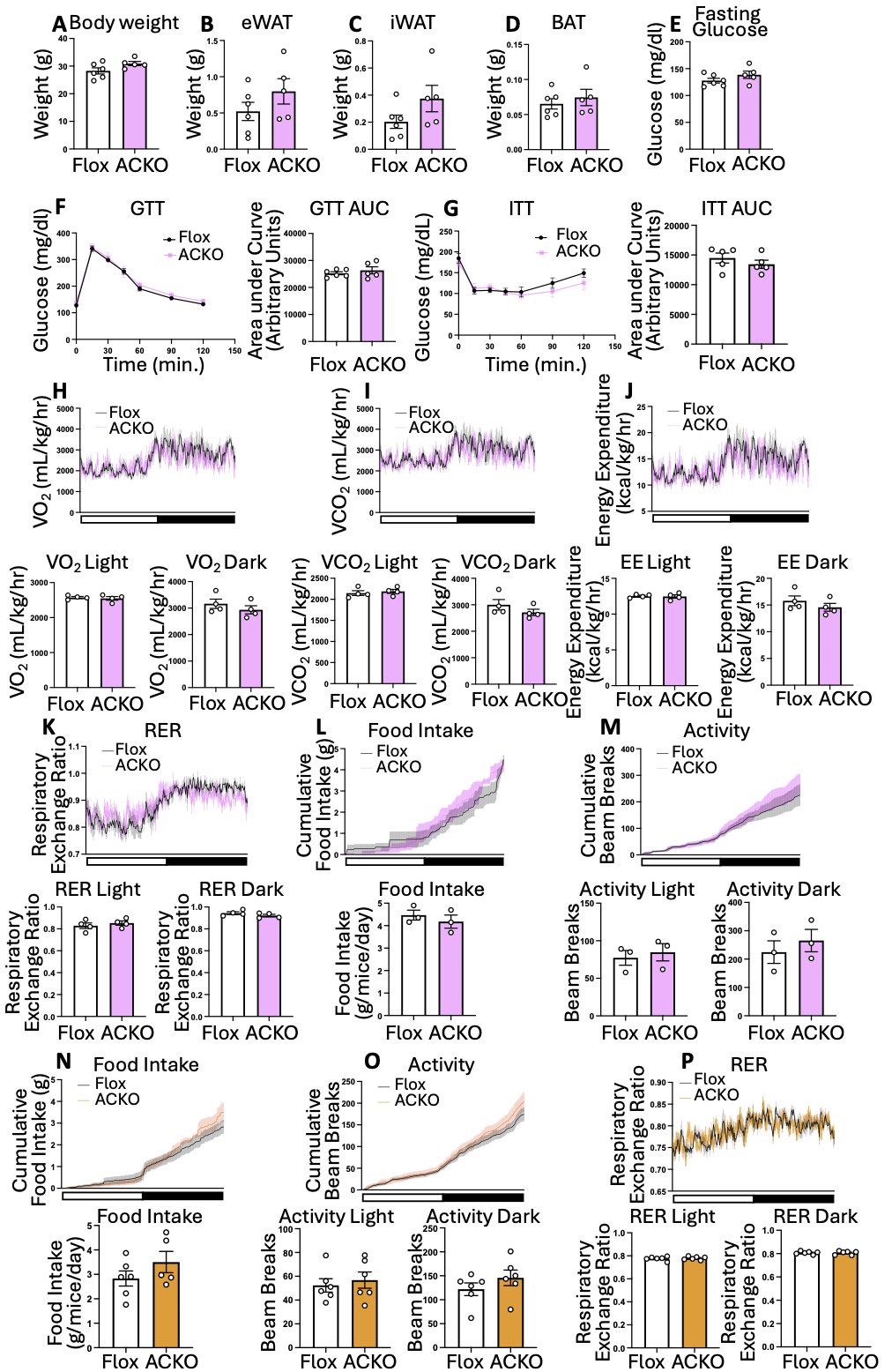


**Supplemental Figure 10. Adipocyte-specific caspase-6 knockout (ACKO) does not affect metabolism in chow diet-fed mice. A-M**. Flox and ACKO mice fed chow diet for 12 weeks. (**A**) Body weight (n=5-6). (**B-D**) Tissue weights (n=5-6): eWAT (**B**), iWAT (**C**), and BAT (**D**). (**E**) Fasting glucose (n=5-6). Two tailed unpaired Student’s *t*-test. (**F**) GTT with AUC quantification (n=5-6). (**G**) ITT with AUC quantification (n=5-6). GTT and ITT: two-way ANOVA followed by Šídák’s-corrected *post hoc* test. **H-M**. Indirect calorimetry of Flox and ACKO mice fed chow diet for 12 weeks (n=4): oxygen consumption rate (**H**), carbon dioxide production (**I**), energy expenditure (**J**). (H-J) ANCOVA analysis with body weight as a covariate. RER (**K**), food intake (**L**), physical activity (**M**). **(K-M)** Two-way ANOVA followed by Šídák’s-corrected *post hoc* test. **N-P**. Indirect calorimetry of Flox and ACKO mice fed HFD for 12 weeks (n=6): food intake (**N**), activity (**O**), and RER (**P**). Two-way ANOVA followed by Šídák’s-corrected *post hoc* test. *p<0.05, **p<0.01, ***p<0.001, ****p<0.0001.
