# Supplementary material for: Caspase‐6 Controls Lipid and Energy Metabolism in Diet‐Induced Obesity
Source: Adv Sci (Weinh). 2026 Apr 13;13(21):e14784. doi: 10.1002/advs.202514784 (PMC13073318; doi:10.1002/advs.202514784)
Supplement: Supplementary file 6 — Supporting File 6: advs73758‐sup‐0006‐DataFile.pdf. [file ADVS-13-e14784-s004.pdf]

1a

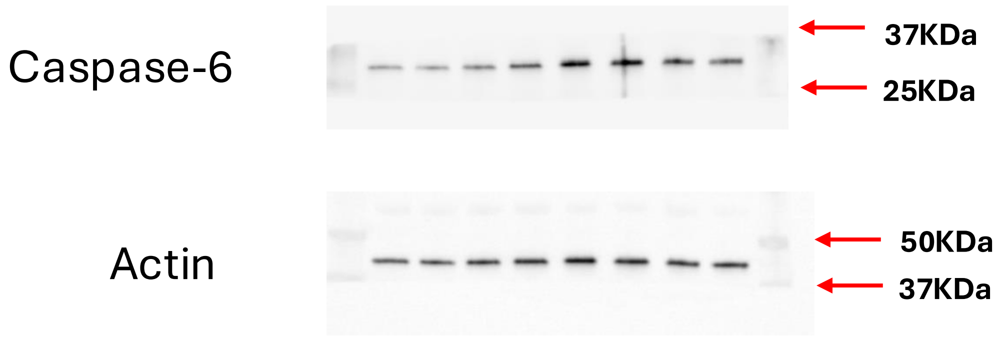

1b

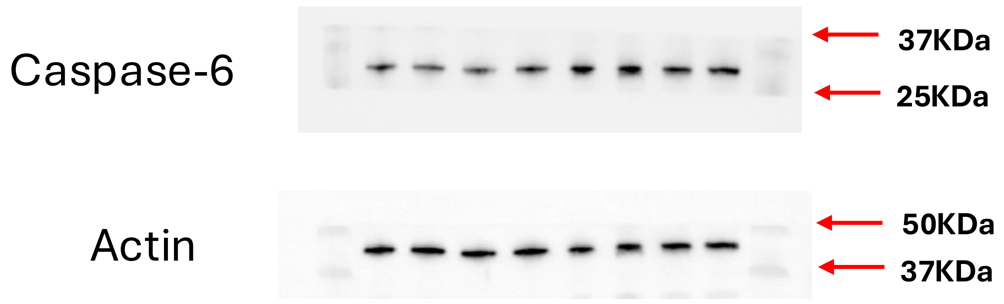

3b

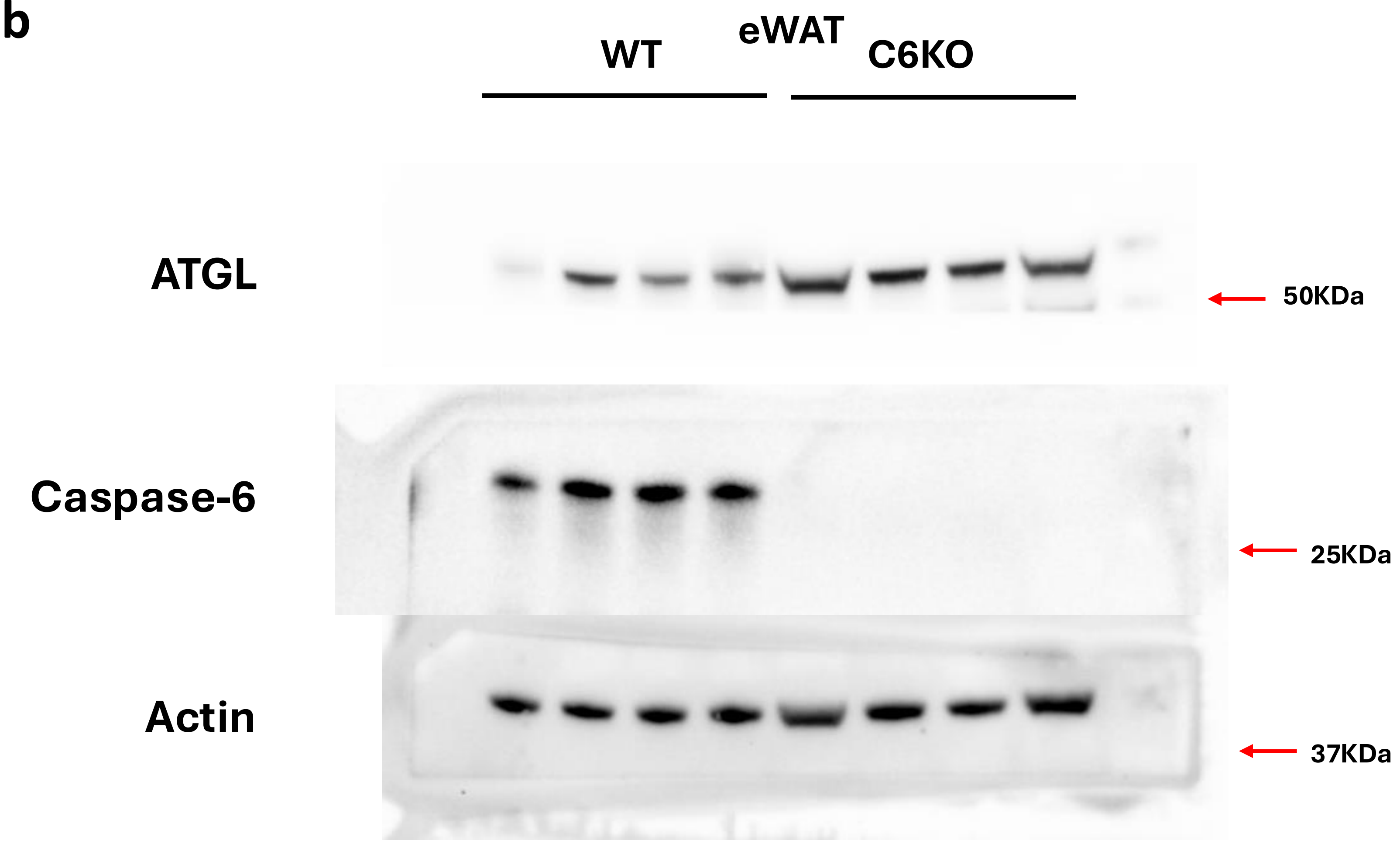

3c

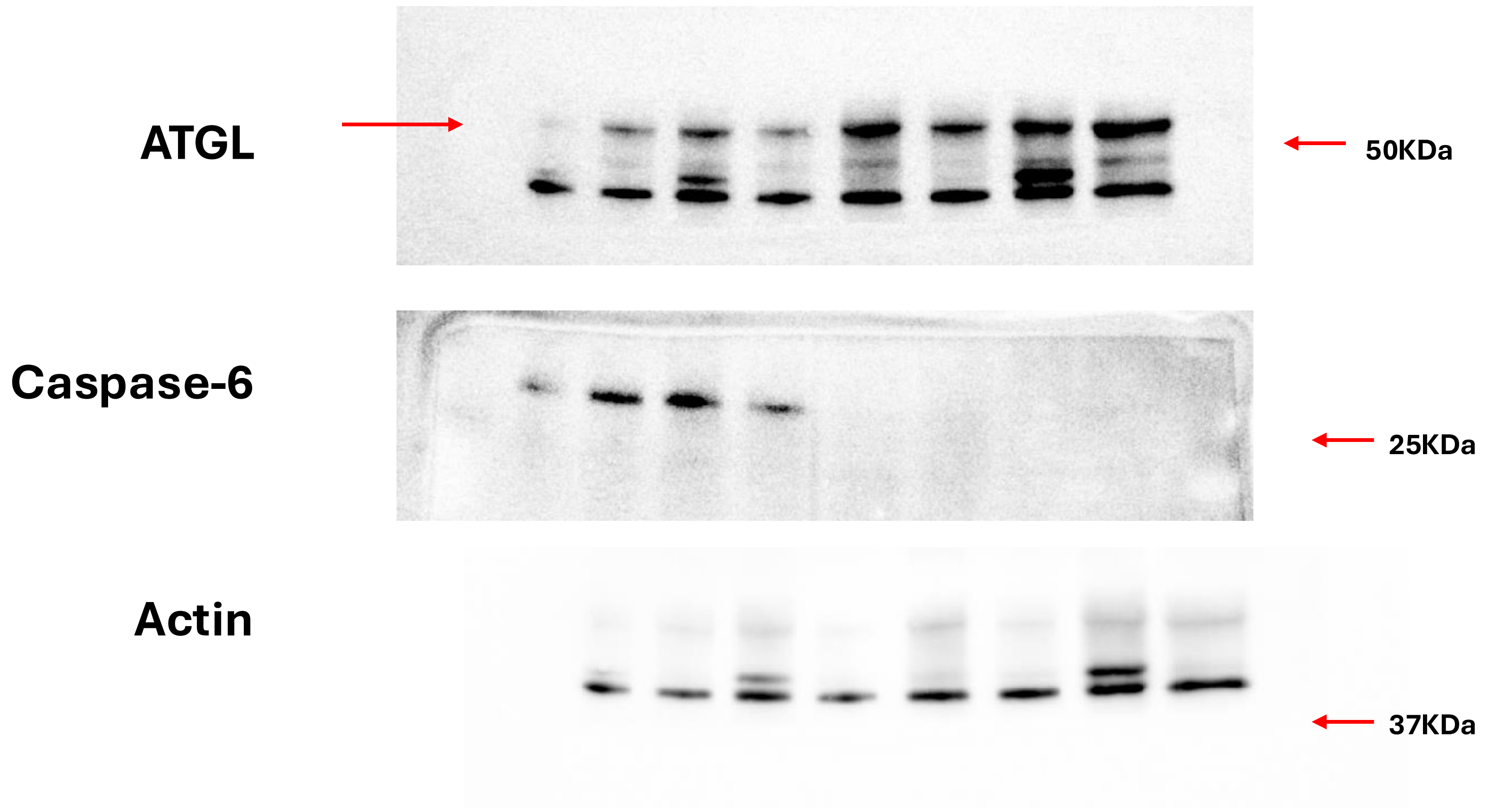

4c

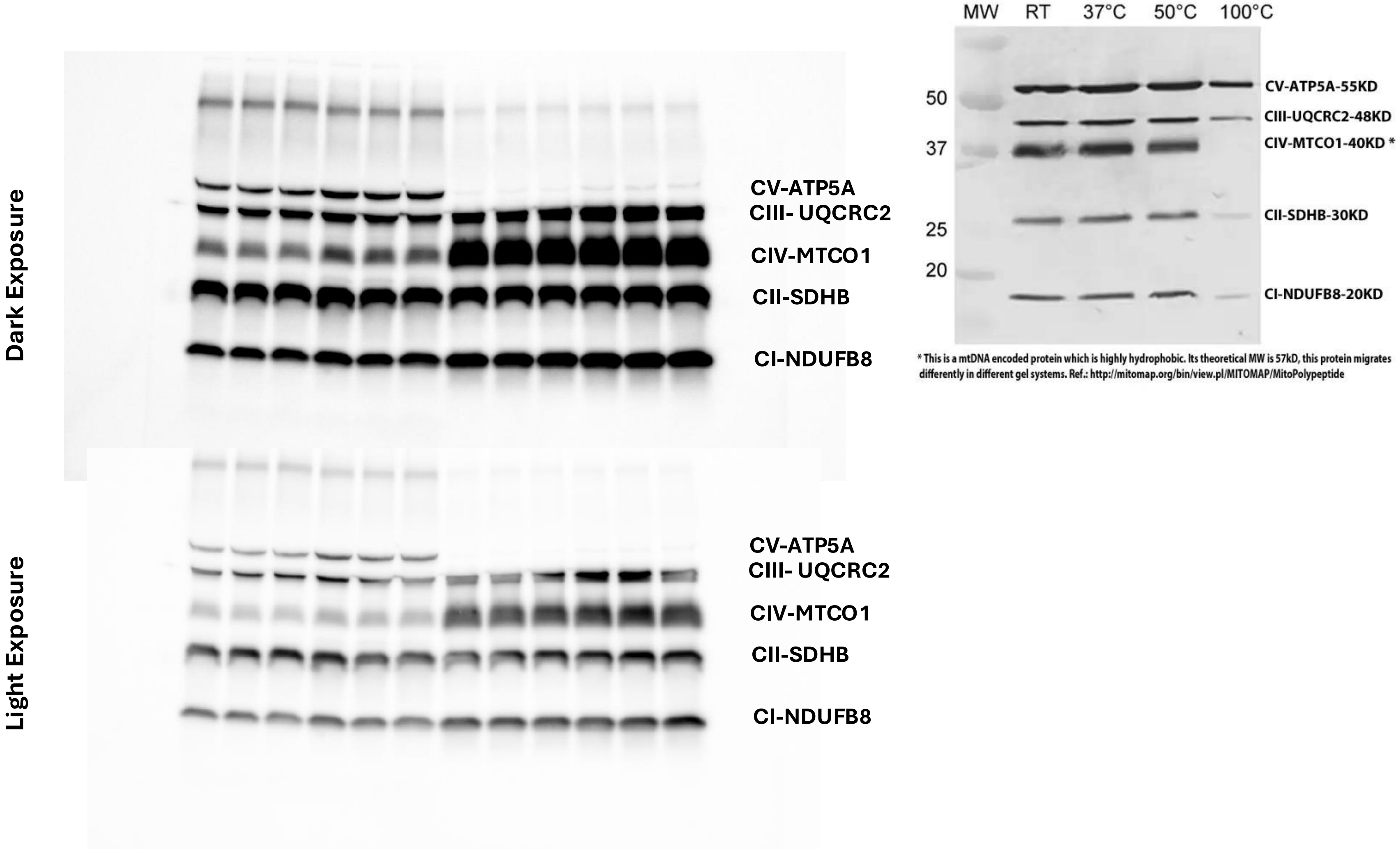
Dark Exposure
CV-ATP5A
CIII- UQCRC2
CIV-MTCO1
CII-SDHB
CI-NDUFB8
Light Exposure
CV-ATP5A
CIII- UQCRC2
CIV-MTCO1
CII-SDHB
CI-NDUFB8
MW
RT
37°C
50°C
100°C
50
37
25
20
CV-ATP5A-55KD
CIII-UQCRC2-48KD
CIV-MTCO1-40KD *
CII-SDHB-30KD
CI-NDUFB8-20KD
* This is a mtDNA encoded protein which is highly hydrophobic. Its theoretical MW is 57kD, this protein migrates differently in different gel systems. Ref.: http://mitomap.org/bin/view.pl/MITOMAP/MitoPolypeptide

5a

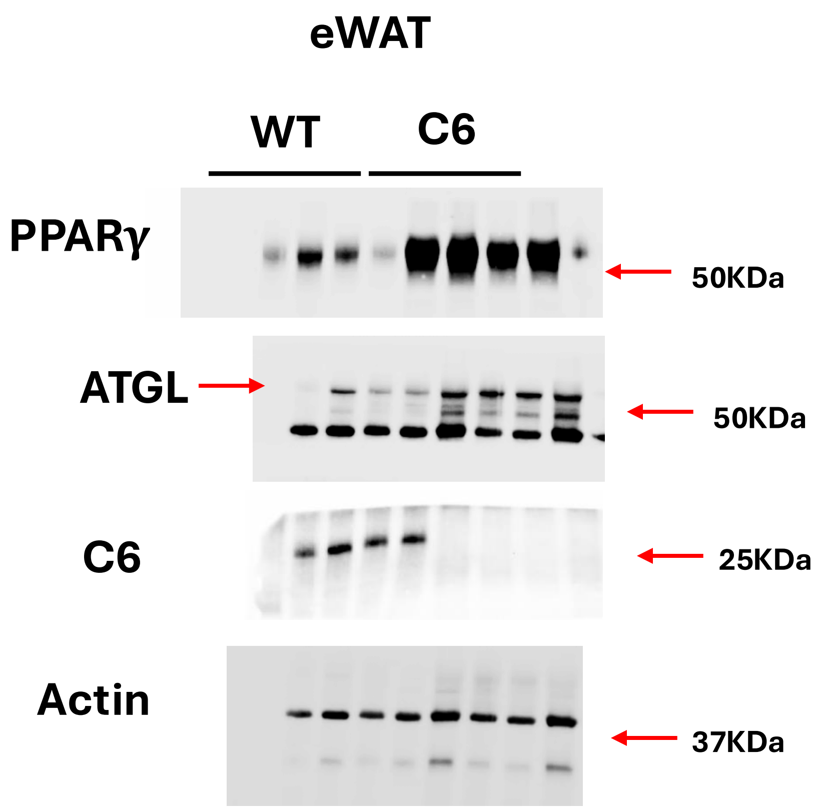

5b

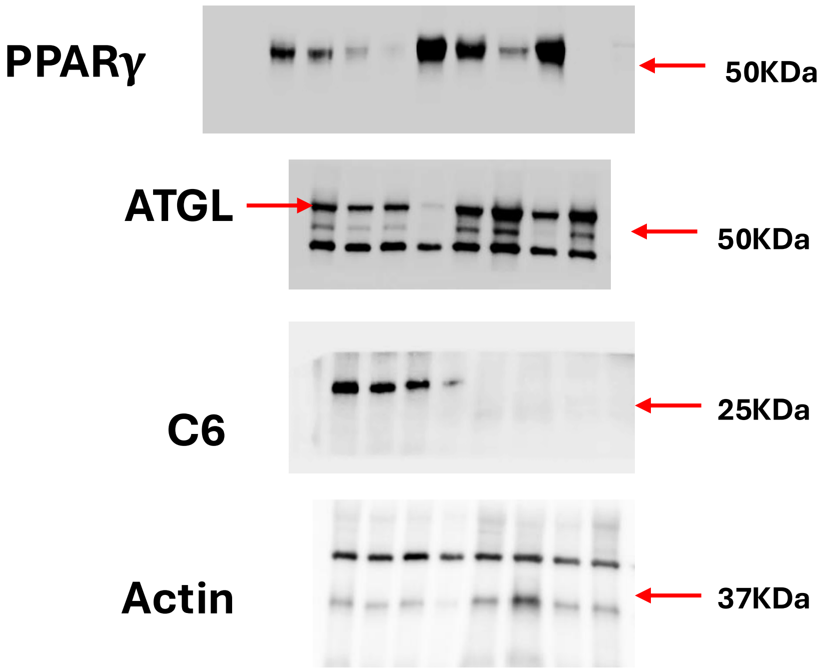

5c

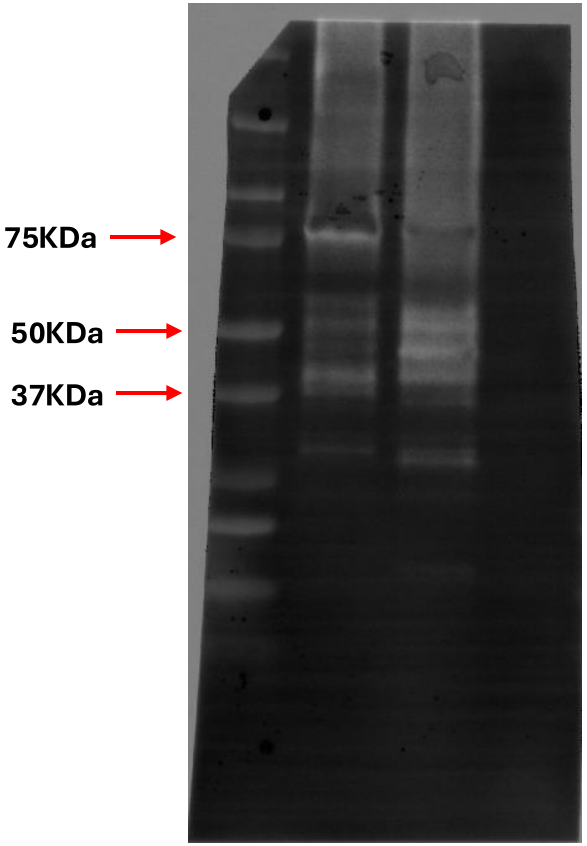
75KDa
50KDa
37KDa

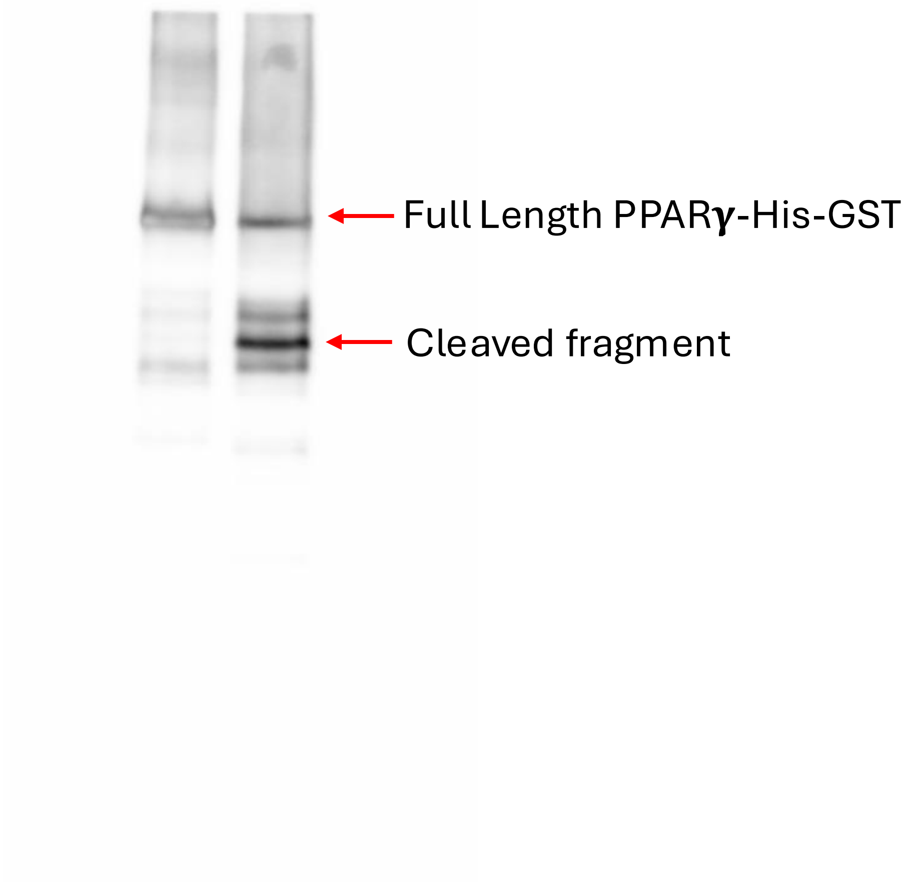
PPARγ-His-GST
Full Length PPARγ-His-GST
Cleaved fragment

5d

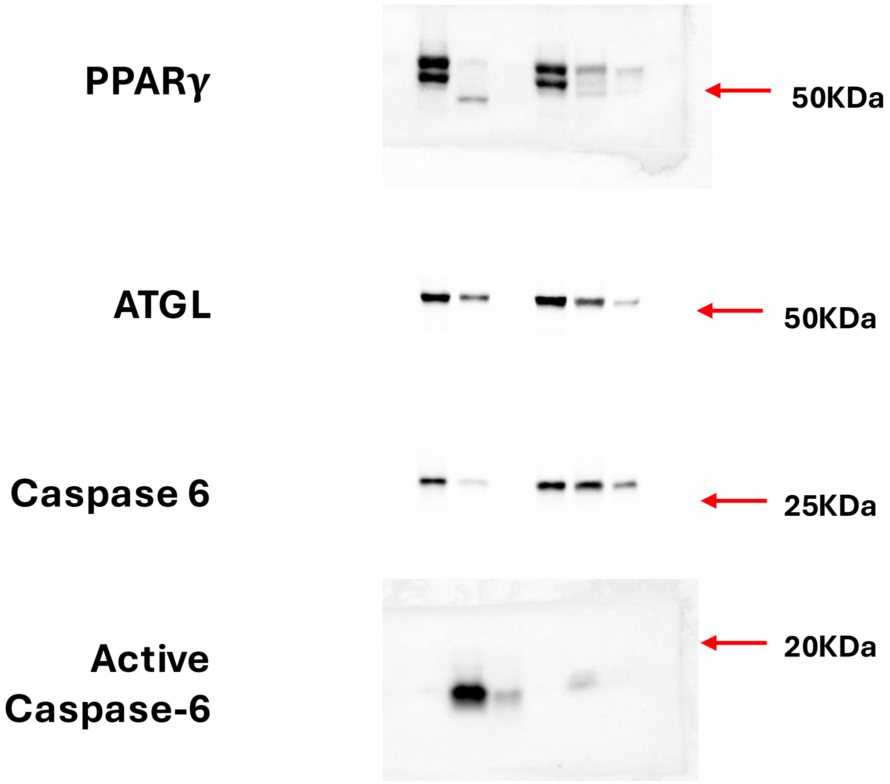

PPARγ
50KDa
ATGL
50KDa
Caspase 6
25KDa
Active
Caspase-6
20KDa

5e

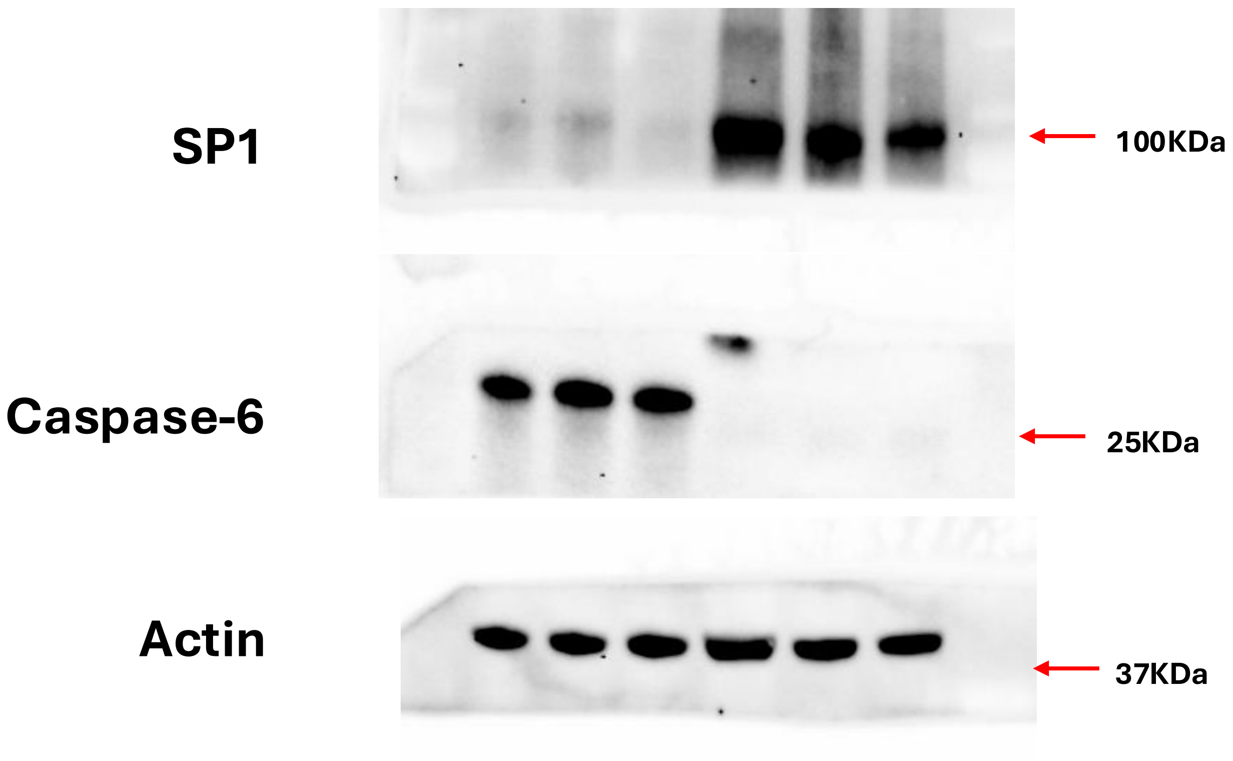

5f

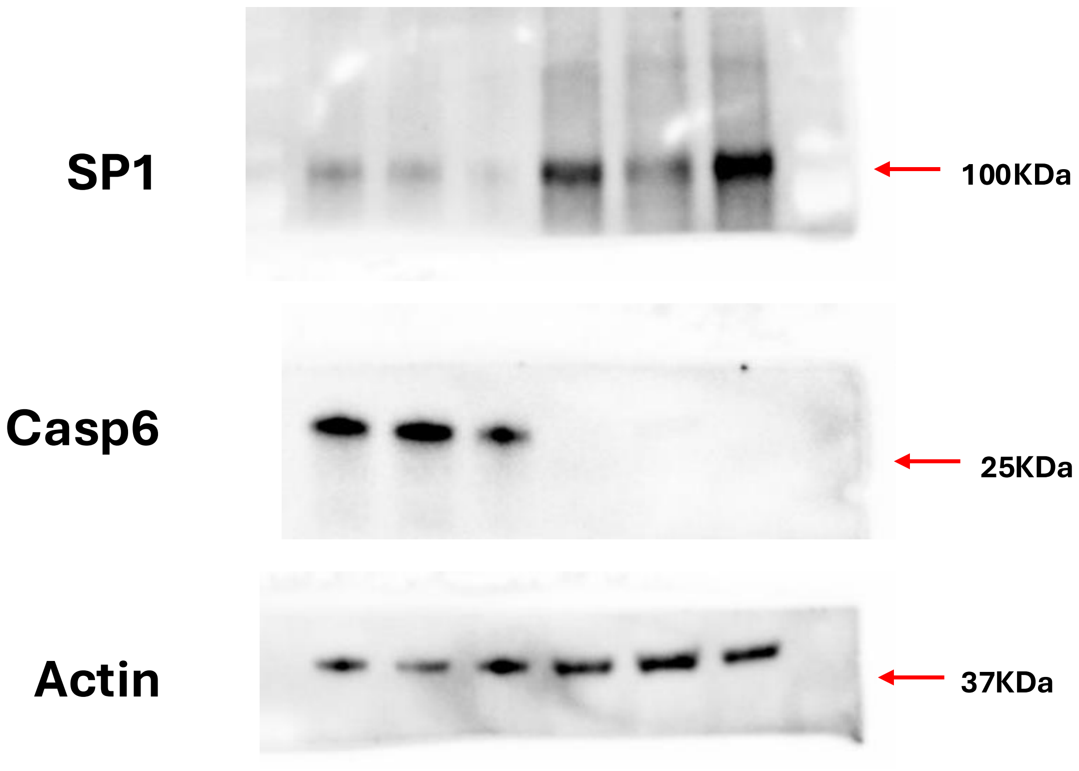

5g

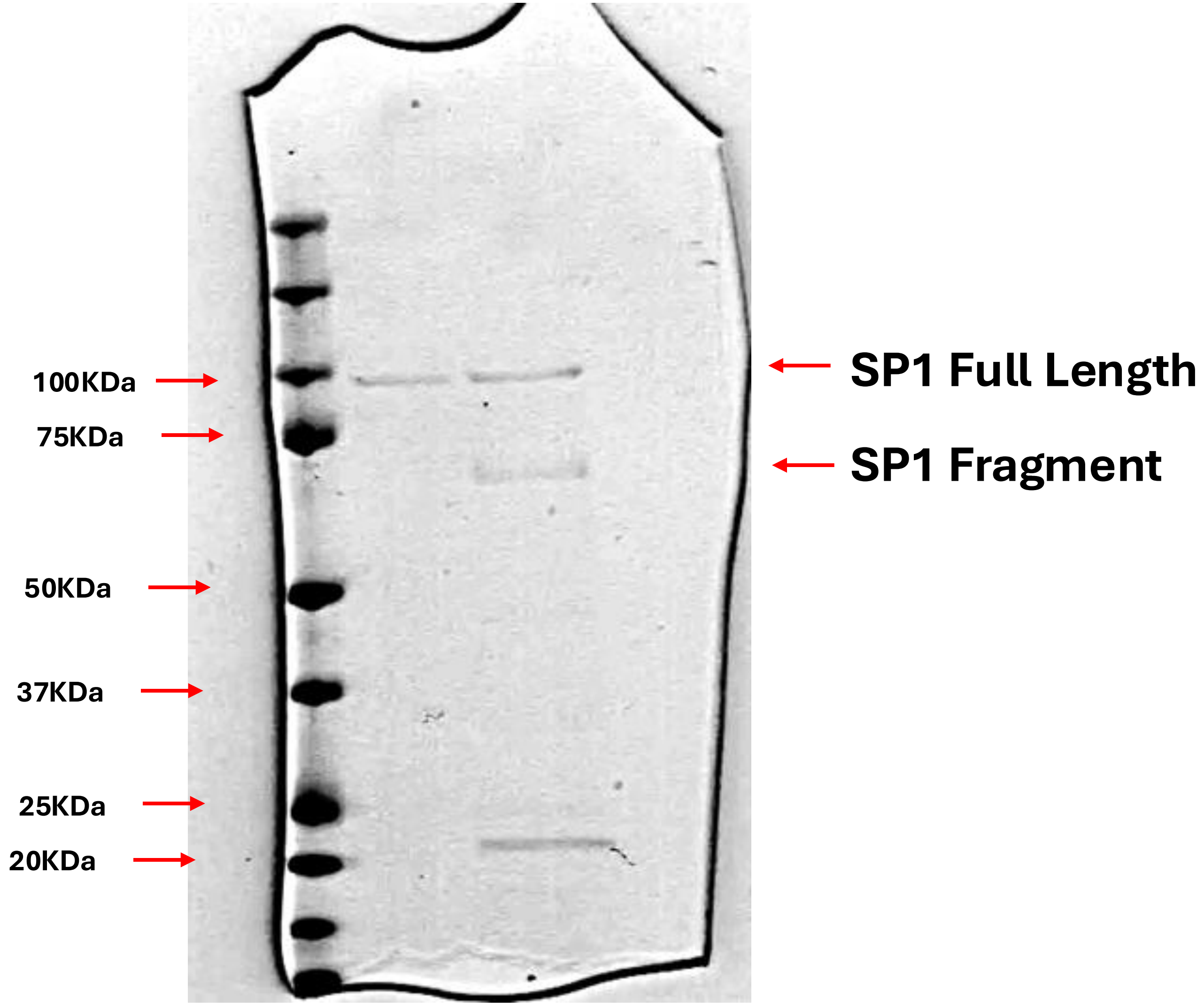

100KDa
75KDa
50KDa
37KDa
25KDa
20KDa
SP1 Full Length
SP1 Fragment

5h

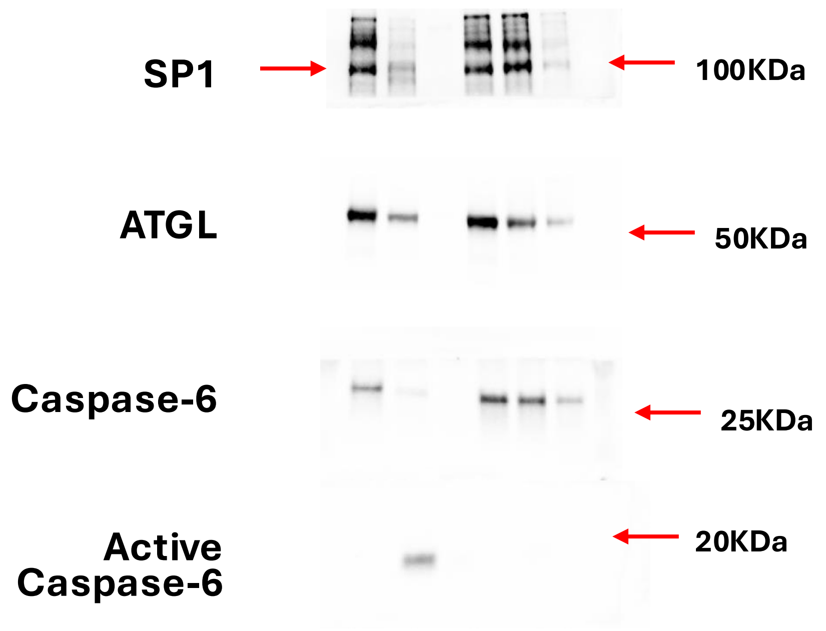
SP1
100KDa
ATGL
50KDa
Caspase-6
25KDa
Active
Caspase-6
20KDa

5i

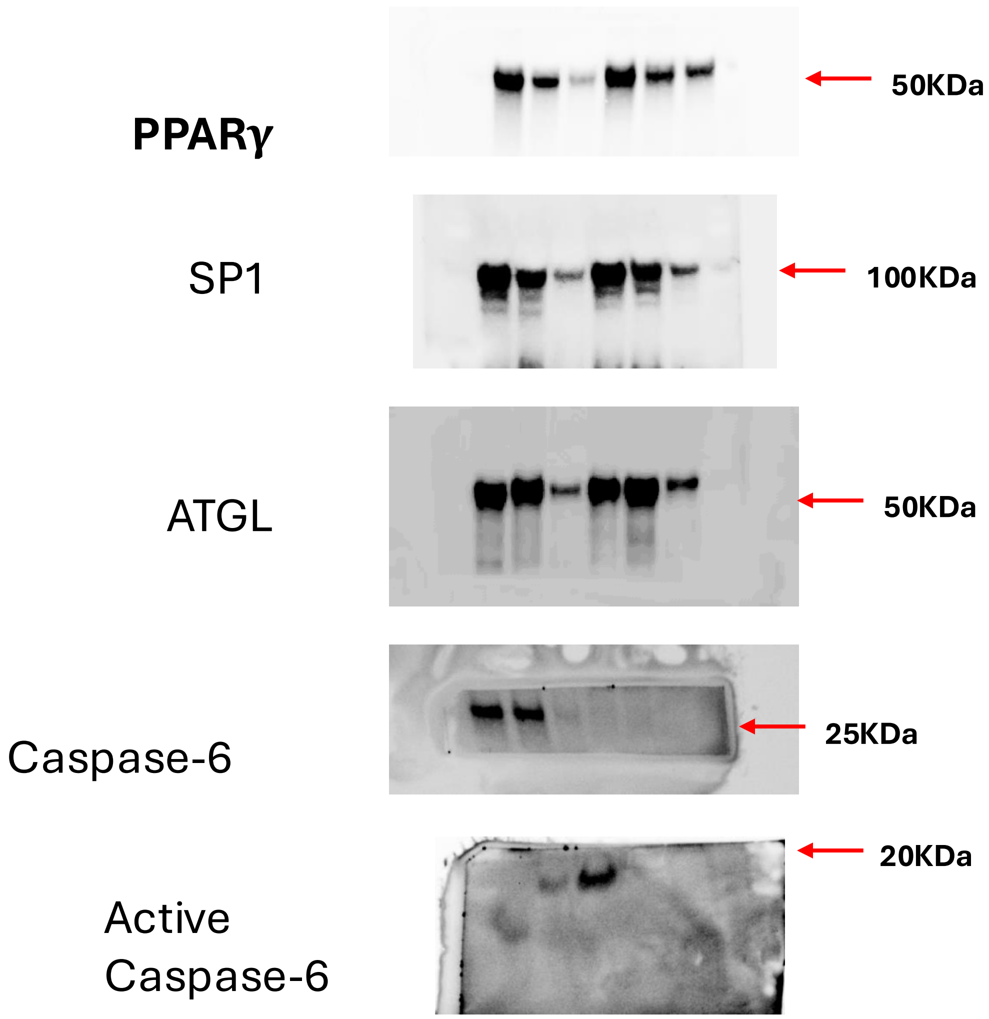
PPARγ
50KDa
SP1
100KDa
ATGL
50KDa
Caspase-6
25KDa
Active
Caspase-6
20KDa

5k

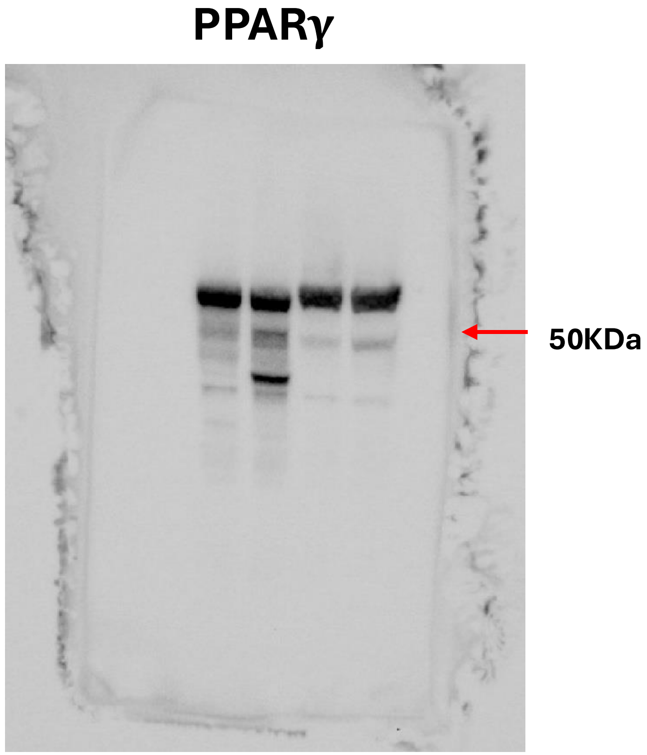

5l

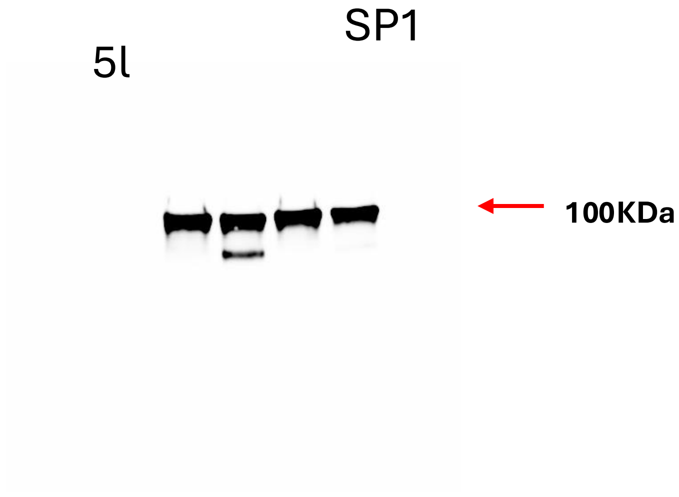

6b

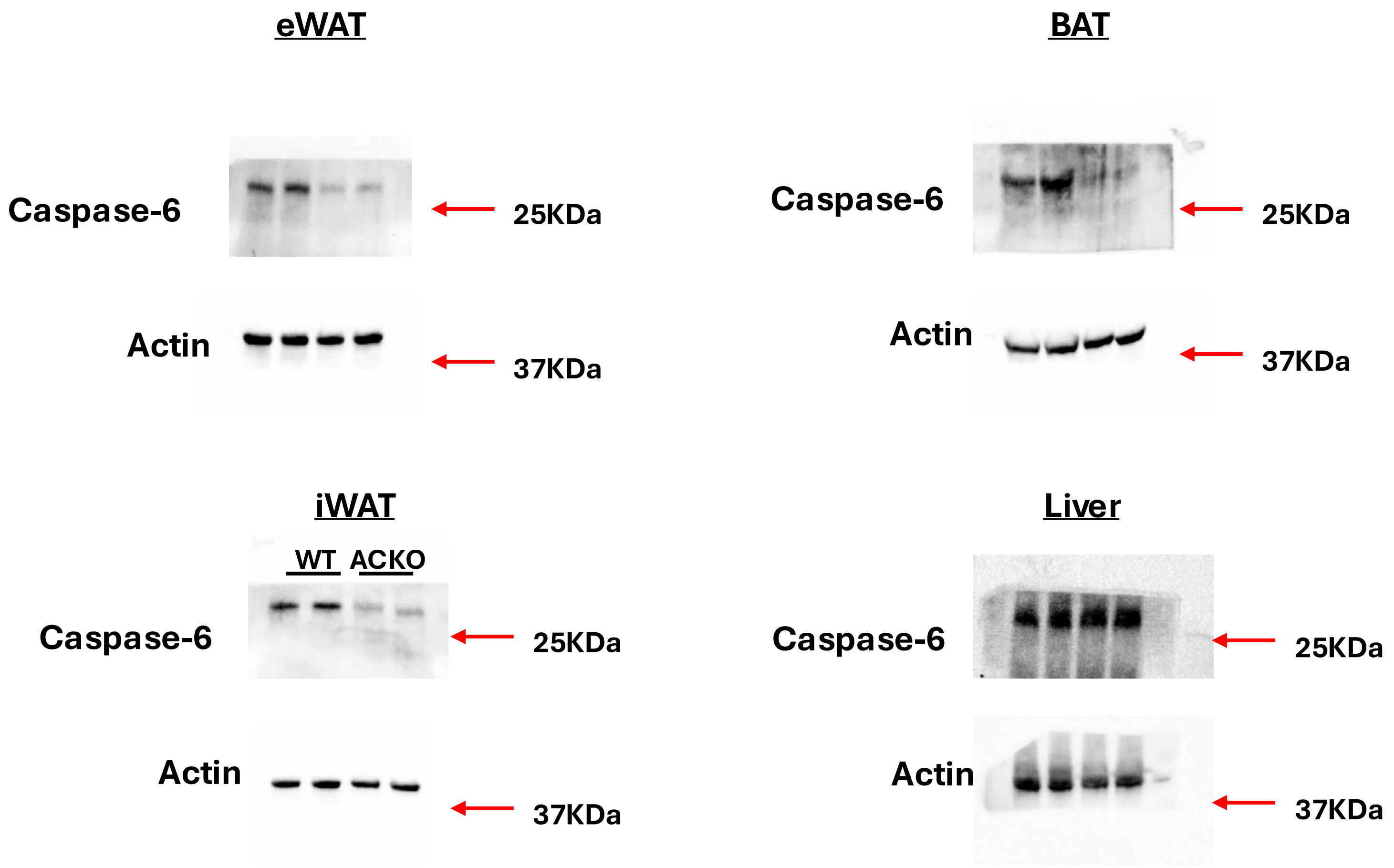
eWAT
Caspase-6
25KDa
Actin
37KDa
BAT
Caspase-6
25KDa
Actin
37KDa
iWAT
WT ACKO
Caspase-6
25KDa
Actin
37KDa
Liver
Caspase-6
25KDa
Actin
37KDa

6l

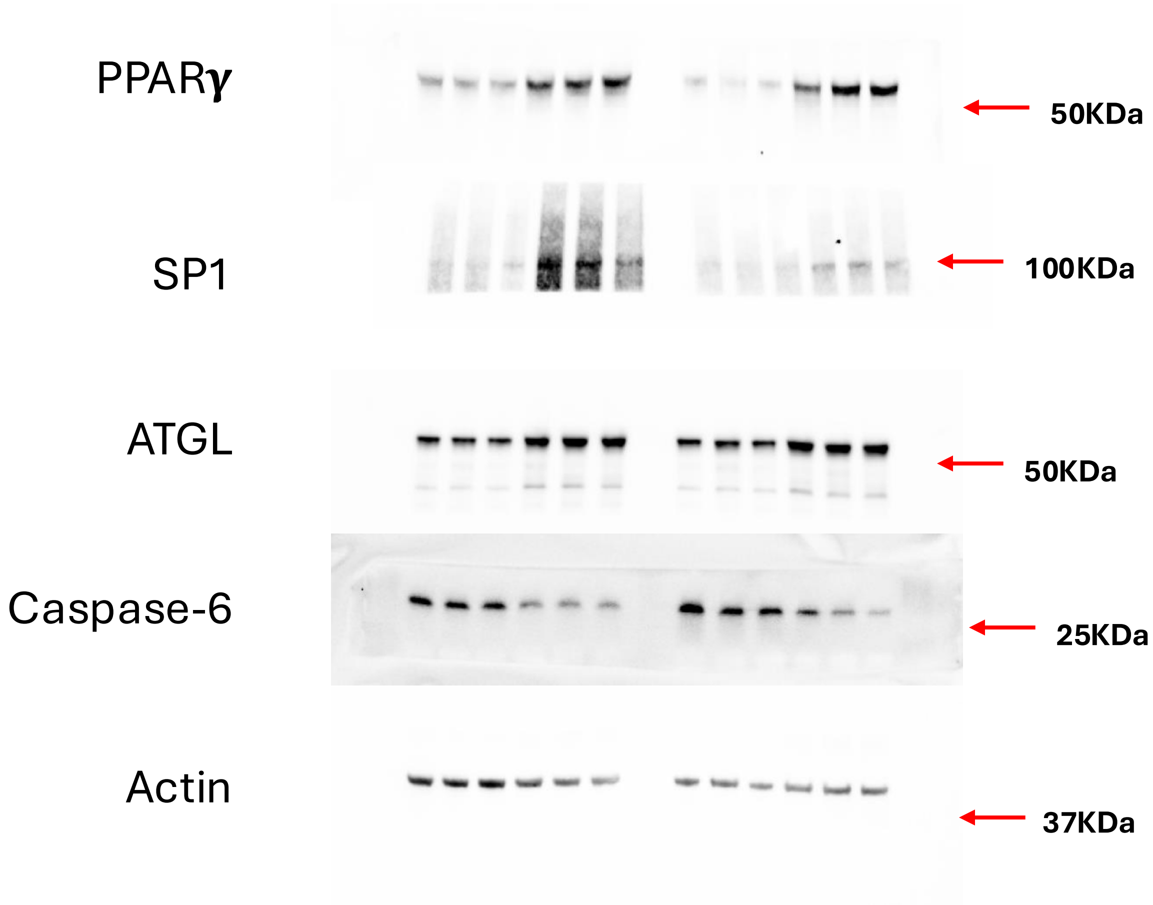
PPARγ
SP1
ATGL
Caspase-6
Actin
50KDa
100KDa
50KDa
25KDa
37KDa

S1A

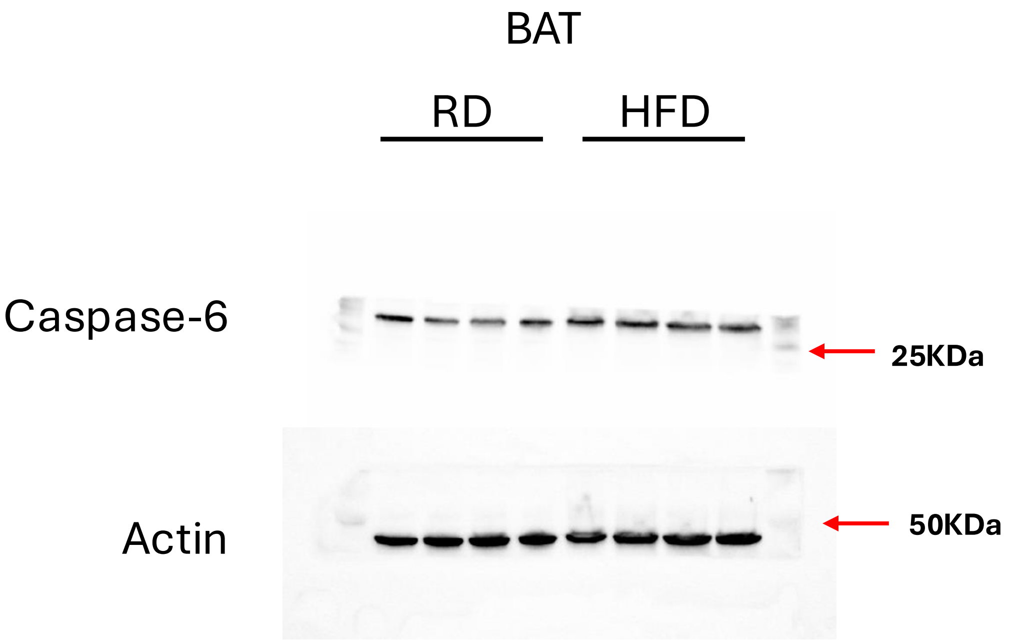

S1B

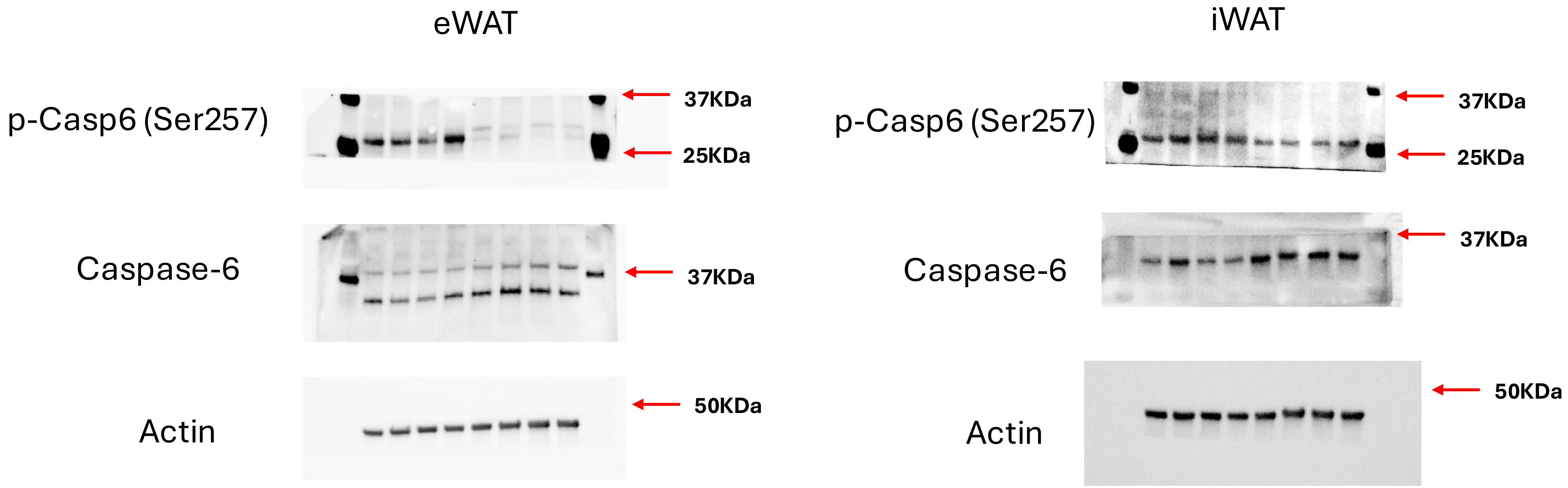
eWAT
iWAT
p-Casp6 (Ser257)
37KDa
25KDa
Caspase-6
37KDa
Actin
50KDa
p-Casp6 (Ser257)
37KDa
25KDa
Caspase-6
37KDa
Actin
50KDa

S1C

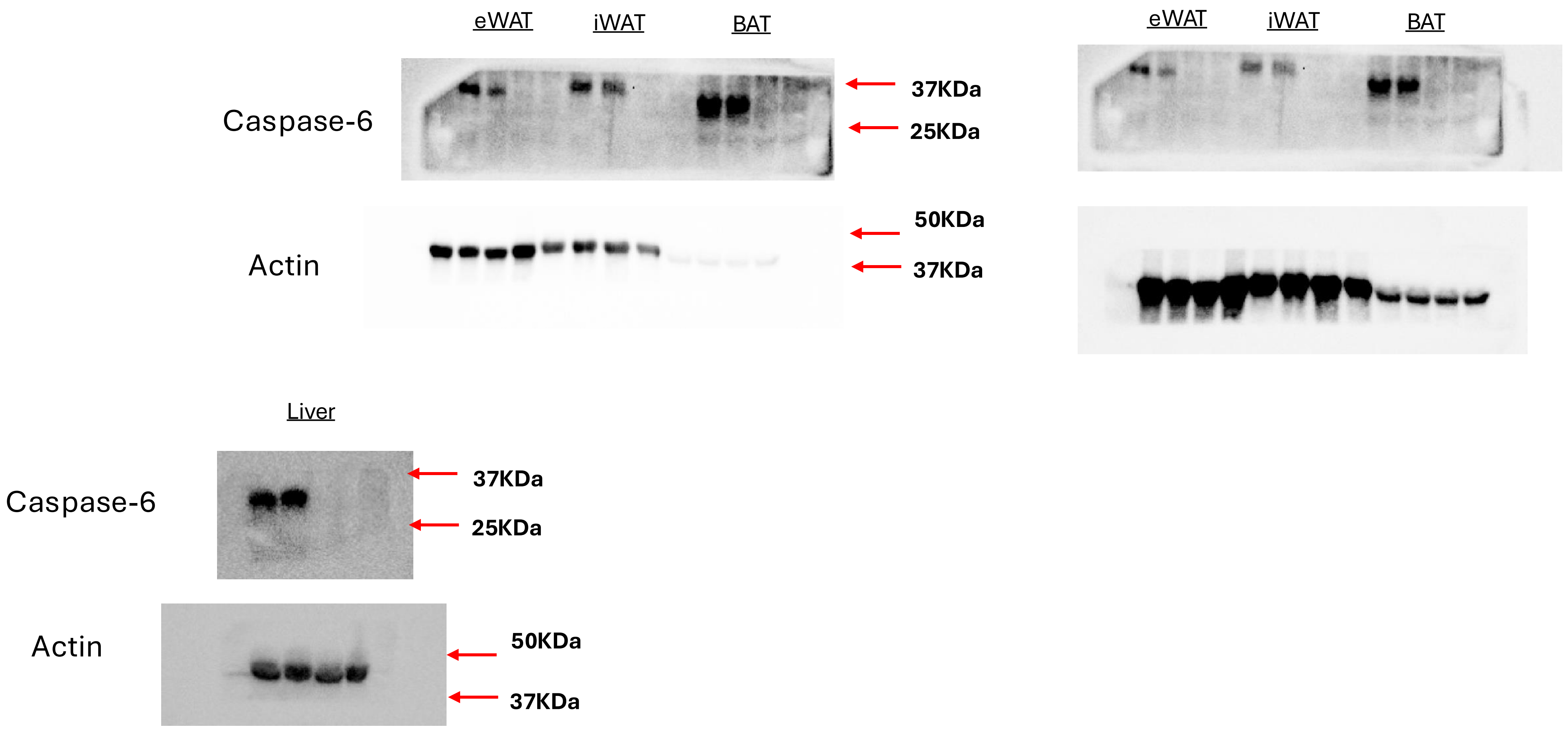
eWAT
iWAT
BAT
Caspase-6
37KDa
25KDa
Actin
50KDa
37KDa
eWAT
iWAT
BAT
Liver
Caspase-6
37KDa
25KDa
Actin
50KDa
37KDa

S9A

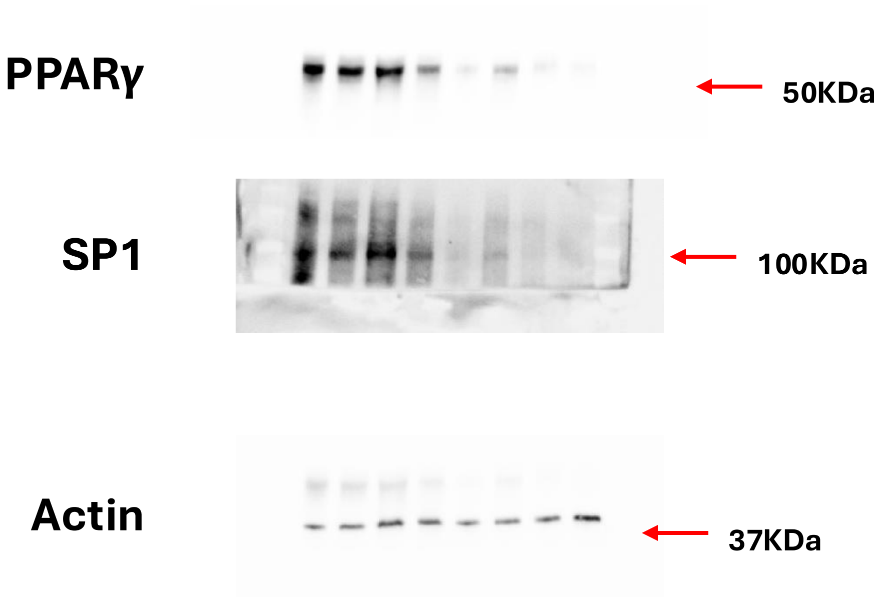

S9B

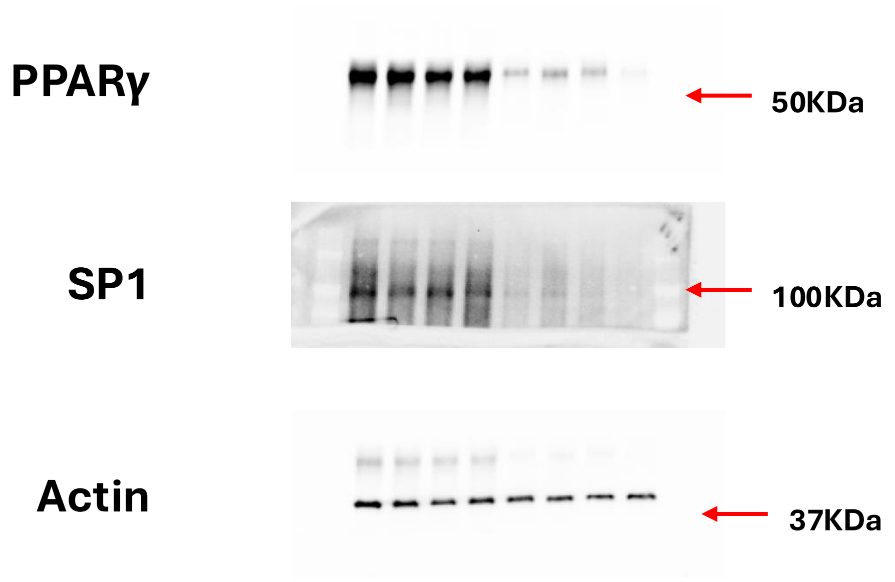

S9C

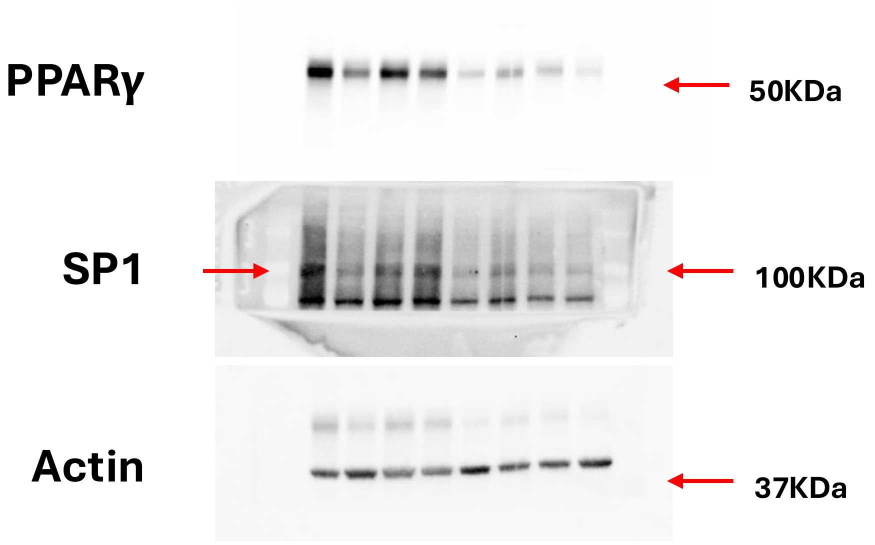

S9G

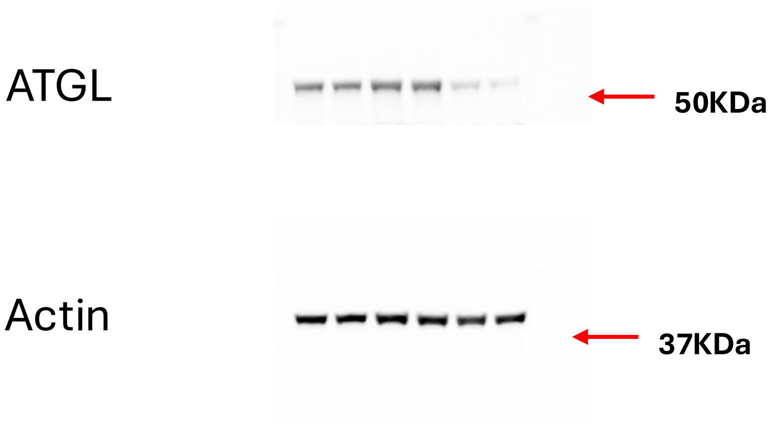

S9I

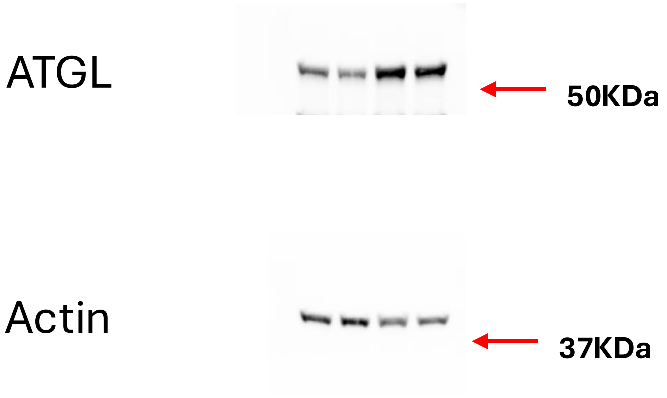

S9Q

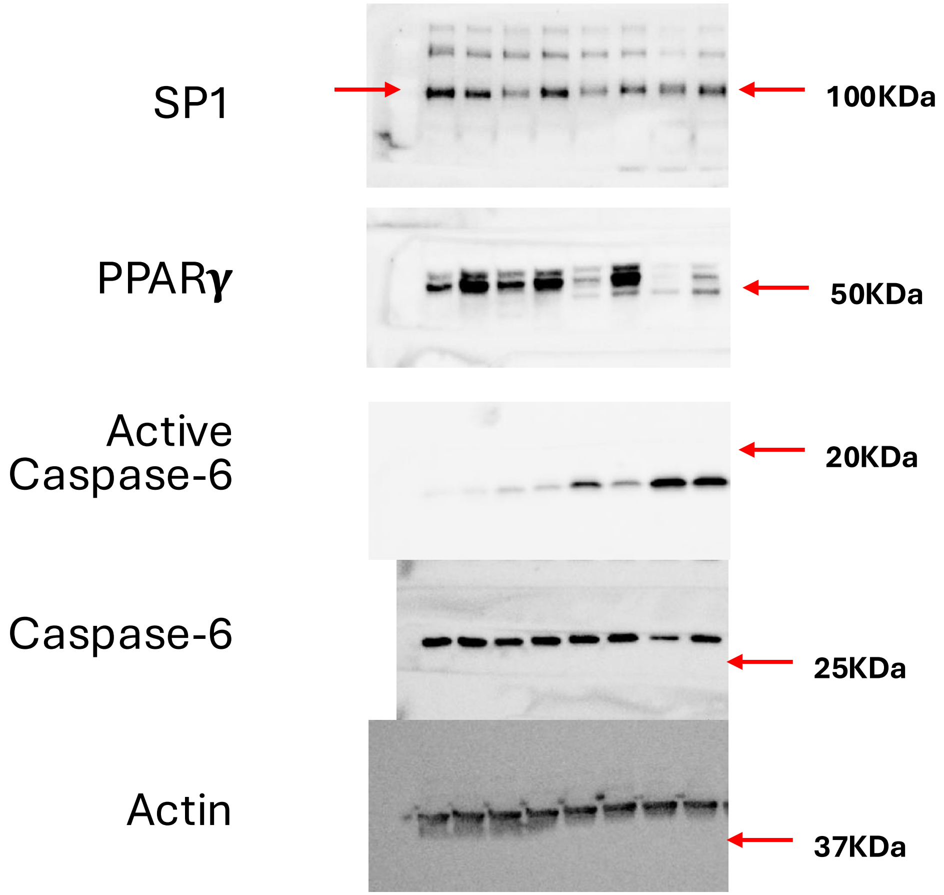

SP1
100KDa
PPARγ
50KDa
Active Caspase-6
20KDa
Caspase-6
25KDa
Actin
37KDa

S9R

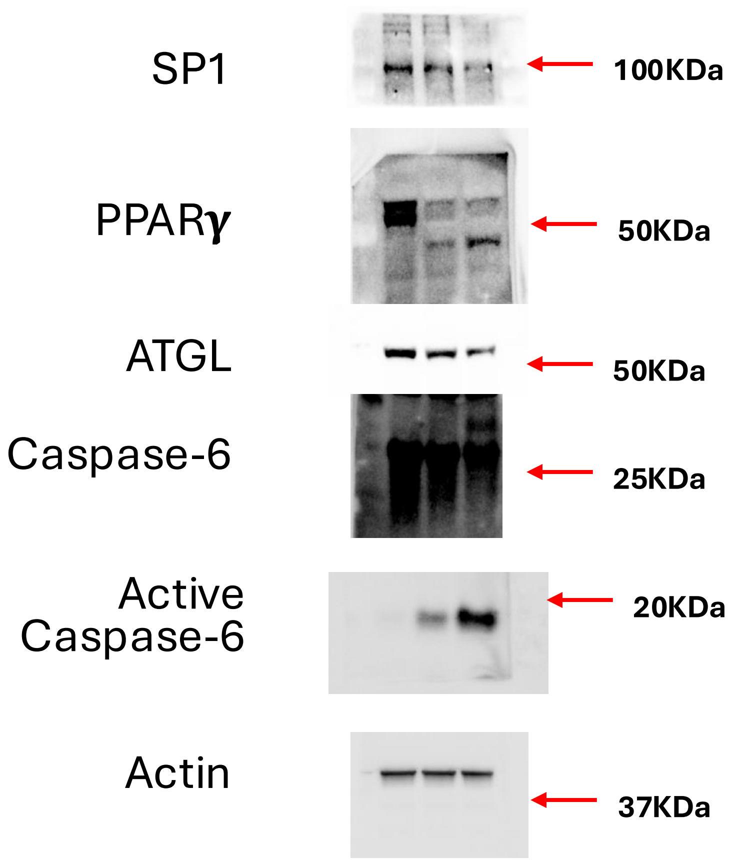
SP1
100KDa
PPARγ
50KDa
ATGL
50KDa
Caspase-6
25KDa
Active Caspase-6
20KDa
Actin
37KDa
